# Novel broadly reactive monoclonal antibody protects against *Pseudomonas aeruginosa* infection

**DOI:** 10.1128/iai.00330-24

**Published:** 2024-12-13

**Authors:** Margalida Mateu-Borrás, Spencer R. Dublin, Jason Kang, Hunter L. Monroe, Emel Sen-Kilic, Sarah J. Miller, William T. Witt, Joshua A. Chapman, Gage M. Pyles, Shreeram C. Nallar, Annalisa B. Huckaby, Evita Yang, Carleena Rocuskie-Marker, Megan E. Grund, Md Shahrier Amin, Slawomir Lukomski, Greg A. Snyder, Krishanu Ray, George K. Lewis, Darrell O. Ricke, F. Heath Damron, Mariette Barbier

**Affiliations:** 1 Department of Microbiology, Immunology, and Cell Biology, West Virginia University 5631 , Morgantown, West Virginia, USA; 2 Vaccine Development Center, West Virginia University 53422 , Morgantown, West Virginia, USA; 3 Department of Pathology, Anatomy, and Laboratory Medicine, West Virginia University 5631 , Morgantown, West Virginia, USA; 4 School of Medicine, Division of Vaccine Research, Institute of Human Virology, University of Maryland Baltimore 12264 , Baltimore, Maryland, USA; 5 Lincoln Laboratory, MIT 57663 , Lexington, Massachusetts, USA; Georgia Institute of Technology, Atlanta, Georgia, USA

**Keywords:** *Pseudomonas aeruginosa*, monoclonal antibodies, anti-microbial resistance, Burkholderia

## Abstract

The incidence of infections attributed to antimicrobial-resistant (AMR) pathogens has increased exponentially over the recent decades reaching 1.27 million deaths worldwide in 2019. Without intervention, these infections are predicted to cause up to 10 million deaths a year and incur costs of up to 100 trillion US dollars globally by 2050. The emergence of AMR bacteria such as the ESKAPEE pathogens, and in particular *Pseudomonas aeruginosa* and species from the genus *Burkholderia*, underscores an urgent need for new therapeutic strategies. Monoclonal antibody (mAb) therapy offers a promising alternative to treat and prevent bacterial infections. In this study, we used peptides from highly conserved areas of the bacterial flagellin to generate monoclonal antibodies capable of broad binding to flagellated Gram-negative bacteria. We generated a broadly reactive IgG2bĸ mAb (WVDC-2109) that recognizes *P. aeruginosa, Burkholderia* sp., and other Gram-negative pathogens of interest. Characterization of the therapeutic potential of this antibody was determined using *P. aeruginosa* as model. *In vitro* characterization of WVDC-2109 demonstrated complement-mediated bactericidal activity and enhanced opsonophagocytosis of *P. aeruginosa*. Prophylactic administration of WVDC-2109 markedly improved survival and outcome in a lethal sepsis model and a sub-lethal murine pneumonia model of *P. aeruginosa* infection, reducing bacterial burden and inflammation. These findings suggest that WVDC-2109 and similar FliC-targeting antibodies could be valuable in preventing or treating diseases caused by *P. aeruginosa* as well as other life-threatening diseases of concern.

**IMPORTANCE:**

Antimicrobial resistance (AMR) costs hundreds of thousands of lives and billions of dollars annually. To protect the population against these infections, it is imperative to develop new medical countermeasures targeting AMR pathogens like *P. aeruginosa* and *Burkholderia* sp. The administration of broadly reactive monoclonal antibodies can represent an alternative to treat and prevent infections caused by multi-drug-resistant bacteria. Unlike vaccines, antibodies can provide protection regardless of the immune status of the infected host. In this study, we generated an antibody capable of recognizing flagellin from *P. aeruginosa* and *B. pseudomallei* along with other Gram-negative pathogens of concern. Our findings demonstrate that the administration of the monoclonal antibody WVDC-2109 enhances survival rates and outcomes in different murine models of *P. aeruginosa* infection. These results carry significant implications in the field given that there are no available vaccines for *P. aeruginosa*.

## INTRODUCTION

Antimicrobial resistance (AMR) has become one of the most important public health challenges worldwide, carrying important clinical and economic consequences. The misuse and abuse of antimicrobials have caused an exponential increase in the number of infections caused by AMR pathogens over the last decades, resulting in an estimated 1.27 million deaths worldwide in 2019 (1). Among these pathogens, *Pseudomonas aeruginosa* poses a particularly challenging problem. *Pseudomonas aeruginosa* is an opportunistic pathogen responsible for a wide range of severe infections, including acute and chronic respiratory infections as well as bloodstream infections (2, 3). It is one of the ESKAPEE pathogens (*Enterococcus faecium, Staphylococcus aureus, Klebsiella pneumoniae, Acinetobacter baumannii, P. aeruginosa, Enterobacter spp*., and *Escherichia coli*), which are the leading cause of life-threatening healthcare-associated infections (HAIs) throughout the world. Notably, multidrug-resistant *P. aeruginosa* alone accounts for 13%–19% of HAIs annually in the United States (4, 5). Bacteria from the genus *Burkholderia* are also notoriously resistant to antimicrobials (6). This genus encompasses many human pathogens such as the select agents *B. pseudomallei* and *B. mallei,* responsible for causing glanders and melioidosis, respectively, and the *B. cepacia* complex, the causative agent of pulmonary infections in cystic fibrosis patients (6). Therefore, new or complementary strategies are needed for infections difficult to treat with conventional antimicrobials.

The use of monoclonal antibodies (mAbs) could represent an alternative to treat or prevent infections caused by those pathogens (7, 8). Although more than 100 mAbs have been approved and licensed, including antibodies for the treatment of RSV (9) and SARS-CoV2 (10), only three antibodies have been authorized by the FDA to treat bacterial infections, and none of them are approved for use against AMR pathogens (11–14). One underlying reason is the elevated cost of production compared to other antimicrobial agents (15). This problem is exacerbated by the specificity of antibodies for select microorganisms, prompting the potential use of mAb cocktails for a broader spectrum of use. To overcome this issue, we propose generating mAbs that bind to an antigen conserved across different Gram-negative organisms such as *P. aeruginosa* and *B. pseudomallei*. In this regard, flagellin or FliC has been considered as a potential candidate for mAb-based therapies.

FliC is the primary protein subunit of the flagellar filament, which contributes to bacterial motility. It is an abundant protein located on the surface of *B. pseudomallei, P. aeruginosa*, and other ESKAPEE pathogens essential for the establishment of infections (16–18). It is highly immunogenic and can elicit an inflammatory response *via* TLR5 activation (19–22). Since flagellin is broadly conserved across *Burkholderia* species, *P. aeruginosa,* and other Gram-negative organisms (23), we hypothesized that targeting antibody development to the conserved regions of the protein will generate antibodies that broadly bind the flagellum of different motile pathogenic species.

In this study, we generated a novel mAb against *P. aeruginosa* and *B. pseudomallei* FliC (WVDC-2109) using hybridoma-based technology. We demonstrated that a synthetic peptide-based strategy of immunization can lead to the generation of mAbs able to broadly recognize both *P. aeruginosa* and *B. pseudomallei* as well as other Gram-negative AMR bacteria. WVDC-2109 contributes to complement-mediated killing of *P. aeruginosa* as well as opsonophagocytic killing by macrophages *in vitro*. We observed that WVDC-2109 has prophylactic activity against *P. aeruginosa* in pre-clinical murine models of acute pneumonia and sepsis. Overall, this study describes a promising mAb candidate for anti-*P*. *aeruginosa* therapy with potential additional applications for other AMR pathogens.

## MATERIALS AND METHODS

### Bacterial strains

All bacterial strains used in this study are listed in Table S1. All *Pseudomonas aeruginosa* strains were grown in Luria-Bertani (LB) broth or on Pseudomonas Isolation Agar (PIA) at 36°C. Clinical isolates of *P. aeruginosa* were described by Burns et al. (24). *Burkholderia* species were grown in LB or on LB agar (LBA). The rest of the bacterial species were cultured in Tryptic Soy Broth (TSB) or on TS agar (TSA) plates at 36°C.

### Peptide design

The sequence of the *B. pseudomallei* FliC protein was analyzed with BlastP (25) against the GenBank database (26) against *P. aeruginosa* and protein multiple sequence alignments were created with Dawn (27) across multiple families of Gram-negative bacteria with 706 sequences for *Vibrionaceae*, 49 for *Xanthomonadaceae*, and 177 for *Pseudomonadaceae* (Data set S1). The variability of aligned protein residues was characterized by the ruby flux.rb program. Candidate peptides of interest were identified from conserved regions identified in the flux results. For structural analyses, the sequence of the *P. aeruginosa* FliC and the *B. pseudomallei* proteins were searched with blastp against the PDB (28) database. Chimera (29) was used to visualize protein structures colored by flux results. Residues highly conserved between all the sequences aligned were identified and cross-compared with previous work characterizing the immunogenicity of the flagellin of *B. pseudomallei* (30, 31). The candidate peptide was selected based on the overlap between sections of high conservation and known immunogenicity. A cysteine residue was added to the N-terminus of the protein to facilitate conjugation with CRM_197._ FliC peptides were generated by life technologies and conjugated to CRM_197_ by FinaBiosolutions (MD, USA).

### Generation of hybridomas

To generate hybridomas, 6-week-old CD-1 female mice (Charles River, strain 022) were immunized intraperitoneally (IP) with 50 µg of the FliC peptide formulated 1:1 with Complete Freund’s adjuvant. At day 21, mice were boosted with 50 µg of the FliC peptide adjuvanted with Incomplete Freund’s adjuvant. Mice were bled *via* sub-mandibular vein 1 week after each injection. The polyclonal antibody response in serum was evaluated by enzyme-linked immunosorbent assay (ELISA) using CRM_197_-conjugated and non-conjugated peptides, *P. aeruginosa* PAO1 and *B. pseudomallei* Bp82-coated plates.

Thirty-four days after priming, the mouse with the highest antibody titers to both *P. aeruginosa* PAO1 and *B. pseudomallei* Bp82 was euthanized with CO_2_. Following euthanasia, blood was collected *via* cardiac puncture, and splenocytes and inguinal lymph nodes were taken and fused with SP2/O-Ag14 myeloma cells (ATCC: CRL-1581) following ClonaCell-HY Hybridoma manufacturer’s instructions. Briefly, 10^7^ myeloma cells were mixed with 10^7^ of the splenocytes and inguinal lymph nodes cell mixture in serum-free Medium B (ClonaCell-HY Medium B, #03802, StemCell Technologies). The mixture was centrifuged and washed three times with an electrofusion medium (Btxpress Cytofusion Medium C, #47001, BTX). After the last wash, cells were placed into a BTX coaxial chamber and subjected to electrofusion using an ECM 2001 Electrofusion and Electroporation System (BTX). After fusion, cells were kept undisturbed in the fusion chamber for 5 min and transferred to a 75 cm^2^ cell culture-treated flask containing Medium C (ClonaCell-HY Medium C, #03803, StemCell Technologies). The process was carried out in duplicate. Flasks were incubated at 37°C, 5% CO_2_, for 16–24 h, after which cells were combined and centrifuged, resuspended with 10 mL of Medium C, and transferred to 90 mL of semi-solid Medium D (ClonaCell–HY Medium D, #03804, StemCell Technologies). After 15 min of incubation at room temperature, 9.5 mL of the suspension was plated into 100 mm × 15 mm Petri dishes using a 10-mL syringe with a 16-gauge blunt-end needle. Plates were incubated at 37°C, 5% CO_2_. Clones representing hybridomas producing monoclonal antibodies were obtained after approximately 10 days. Clones were then individually transferred into 96-well tissue culture-treated plates containing Medium E (ClonaCell-HY Medium E, #03805, StemCell Technologies). After 3–4 days, supernatants of the hybridoma cells were removed and tested by ELISA for antigen specificity against non-conjugated FliC peptides. Positive hybridomas were selected and transferred to 24-well plates containing Medium E. Once confluent, hybridoma supernatants were tested again by ELISA for their reactivity against non-conjugated FliC peptide, *P. aeruginosa* PAO1 and *B. pseudomallei* Bp82. Positive hybridomas were selected and expanded into 50% Medium A (ClonaCell-HY Medium A, #03801, StemCell Technologies) and 50% of Dulbecco’s Modified Eagle Medium (DMEM) (Thermo Fisher Scientific, #MT10013CM) supplemented with 10% vol/vol fetal bovine serum (FBS) (Gibco, #10437028) and 1% vol/v Penicillin-Streptomycin Solution (P/S) (Corning cellgro, #30-001-Cl).

### ELISA binding assays

ELISAs were performed by methods previously described (32). Briefly, 96-well microtiter plates were coated overnight at 4°C with different antigens: 1 µg/mL of CRM-conjugated FliC peptide; 1 µg/mL of non-conjugated FliC peptide; 1 µg/mL of recombinant FliC from *P. aeruginosa* or *B. pseudomallei;* or 2–5 × 10^7^ CFU of the pathogens of interest grown overnight in LB or TSB. Plates were washed three times with 0.05% Tween in PBS (PBS-T) and blocked with 2% wt/vol bovine serum albumin (BSA) in PBS overnight at 4°C. Hybridoma supernatant or purified WVDC-2109 were serially diluted in blocking buffer and incubated for 2 h at 37°C. After four washes with PBS-T, horseradish peroxidase (HRP)-conjugated mouse anti-IgG (Novus Biologicals, #NBP1-75130) was added to the plate at a dilution of 1:2,000 for 1 h at 37°C. Finally, a 1:1 mixture of TMB substrates A (Biolegend, #77247) and B (Biolegend, #77248) was used as a substrate. After 15 min of incubation, the absorbance was measured at 450 nm using SpectraMax i3 spectrophotometer (Molecular Devices LLC). Antibody titers were calculated as the inverse of the last dilution factor at the point at which the absorbance exceeded two times the absorbance of the negative control.

### Isotype determination

The antibody class and subclass and the light chain type were determined using PierceTM Mouse Antibody Isotyping Kit (#26178, Thermo Scientific), following the manufacturer’s instructions. Briefly, the supernatant of the selected hybridomas was diluted 1:10 with the Sample Diluent, and 150 µL of the diluted sample was inserted in each cassette. After 5 min, color bands appeared, and results were evaluated.

### Recombinant flagellin purification

The *fliC* genes from *B. pseudomallei* strain Bp82 and *P. aeruginosa* PAO1 were amplified and cloned into pHERD plasmid (33) with a polyhistidine-tag sequence added. *FliC* genes were expressed in *E. coli* E. cloni Electrocompetent cells (Lucigen), as polyHis-tag FliC protein. Bacterial cells were grown in LB supplemented with Carbenicillin 100 µg/mL. Cells were grown to mid-log phase and induced with 0.5% l-arabinose for 6 h. Cells were pelleted and lysed on ice with 1× PBS containing 160 µg/mL lysozyme (Sigma, L6876), 200 µg/mL DNase I (Sigma, DN25), 1 mM magnesium chloride, and Halt protease inhibitor cocktail (Thermo Scientific, 78430). The extraction was sonicated for 1 min on ice and centrifuged for 10 min at 10,000 × *g*. Proteins were purified from the cell culture supernatant using a histidine column (HisPurCobalt Purification Kit, Fisher Scientific, #90092), following the manufacturer’s instructions. Recombinant FliC purity was confirmed by Coomassie gel staining.

### Monoclonal antibody purification

The hybridoma cell line producing WVDC-2109 was cultured in 50% Medium A and 50% DMEM as indicated above. When confluent, cells were transferred into the fresh medium by centrifugation at 300 *× g* for 5 min. Supernatants were collected, centrifuged for 5 min at 300 *× g,* and filtered with a 0.2 µm pore-size filter to remove cellular debris. WVDC-2109 was purified by affinity chromatography with a GE AKTA Pure Chromatography System by using Protein A (Cytiva HiScreen Fibro PrismA Column) followed by size-exclusion chromatography (Superdex 200 Increase HiScale 16/40 Column). The eluted antibody was dialyzed in PBS using a dialysis membrane of 10K molecular weight cut-off (Thermo Scientific, #88245) for 24 h followed by a 5-h dialysis with 50% Glycerol in PBS and preserved at −20°C until needed. To avoid potential toxicity during murine experiments, prior to use, the excess glycerol was removed using a 10-kDa Amicon Ultra-2 Centrifugal Filter Unit (EMD Millipore, #UFC501024).

### Western blot analysis

Bacteria of interest were grown in PIA, LBA, or TSA plates overnight at 37°C, swabbed into sterile PBS, incubated on ice for 30 min, and lysed using sonication. *B. pseudomallei* and *B. thailandensis* whole-cell lysates (WCL) were obtained by chemical lysis as previously described (34). When necessary, cell lysates were treated with proteinase K (1.3 mg/mL final concentration) or with sodium periodate (NaIO_4_) (13.3 mM final concentration) for 4 h at 37°C. Protein concentration was determined using Pierce BCA Protein Assay Kit (Thermo Fisher, #23225). Recombinant FliC protein from *P. aeruginosa* and *B. pseudomallei* was purified as described above. Protein samples were mixed with 4× Laemmli Sample Buffer (Bio-Rad, #1610747) and Thermo Scientific Bond-Breaker TCEP Solution (Thermo Scientific, #77720) and heated at 95°C for 10 min. Depending on the analysis, 3–10 µg of protein were loaded into protein gels (Bio-Rad, #4569033). Gels were transferred onto rehydrated 0.2 µm PVDF membranes (Bio-Rad, #1704272) using a Trans-Blot Turbo Transfer System (Bio-Rad). Membranes were blocked for 1 h in 5% wt/vol skim milk in PBS-T and incubated overnight at 4°C with WVDC-2109 mAb at a concentration of 5 µg/mL in blocking solution. Next, blots were treated for 1 h at room temperature with an anti-IgG secondary antibody conjugated to HRP (EMD Millipore, #AP127P) at a concentration of 1:5,000 in blocking solution. Finally, membranes were developed using ECL Western Blotting Substrate (Thermo Scientific, #32209). Chemiluminiscent signal was detected using the Chemidoc Touch Imaging System (Bio-Rad, #1708370). Images were visualized and analyzed with Image Lab software version 6.1.0 (Bio-Rad laboratories).

Native PAGE for detection of WVDC-2109 binding to *P. aeruginosa* strains was performed using WCL or its supernatant portion. Briefly, bacteria were grown in LB until reaching the logarithmic phase, centrifuged, and the cell pellet was resuspended to an OD_600_ of 10 per mL in PBS containing 1 mM PMSF. Cells were frozen at −80°C overnight and sonicated until the OD_600_ dropped to 1 per mL. Part of this lysate was used as WCL and the other half part was centrifuged at 12,000 × *g* and the supernatant was used. Samples were prepared by mixing equal volumes of loading buffer with WCL or supernatant. Membranes were blocked as indicated before and incubated overnight with WVDC-2109 125 or 625 ng/mL. An anti-mouse IgG conjugated with IR-800 (LICOR Odyssey) diluted 1:15,000 in blocking buffer was used as a secondary antibody. The signal was detected using a LICOR Odyssey imager.

### Immunogold labeling and transmission electron microscopy

Immunogold labeling and transmission electron microscopy (TEM) were performed as previously described (35), with some modifications. Briefly, formvar-coated copper grids (300 mesh) were disposed of on drops containing overnight *P. aeruginosa* PAO1 culture for 45 min. Grids were washed in PBS and fixed in 2.5% paraformaldehyde. After 20 min, grids were washed three times in PBS and blocked in 5% BSA in PBS for 35 min. Grids were washed and incubated with 100 µg/mL of an isotype control mAb (BioXcell, #BE0086), WVDC-2109, or PBS. After three washes with blocking solution, grids were incubated with goat anti-mouse IgG conjugated to 10 nm colloidal gold (Sigma-Aldrich, #G7652) diluted 1:50 with blocking solution for 45 min. Grids were then washed three times in blocking solution, followed by PBS and miliQ-water, and dried completely on Whatman filter paper (Cytiva, #1001085). Finally, grids were analyzed using a TEM (JEOL JEM-2100) equipped with a Gatan OneView Camera.

### TLR5 assays

TLR5 activation was analyzed using HEK-Blue-hTLR5 cells (InvivoGen, #hkb-htlr5) that express the secreted embryonic alkaline phosphatase (SEAP) reporter gene under the control of NF-κB and AP-1. Cells were cultivated in DMEM with 4.5 g/L glucose supplemented with 10% FBS (vol/vol), 1% P/S (vol), 100 µg/mL Normocin (InvivoGen, #ant-nr-1), 30 µg/mL Blasticidin (InvivoGen, #ant-bl-1), and 100 µg/mL Zeocin (InvivoGen, #ant-zn-1) until reaching 70%–80% of confluence. On the day of the experiment, cells were washed with PBS and detached from the flask. A cell suspension of 1.4 × 10^5^ cells/mL was prepared in HEK-Blue-Detection medium (InvivoGen, #hb-det).

To test the ability of WVDC-2109 to block flagellin-mediated TLR-5 activation, samples were prepared by incubating different concentrations of recombinant purified FliC from *P. aeruginosa* or *B. pseudomallei* (0–100 ng/mL) with different concentrations of WVDC-2109 (0–100 µg/mL). After 90 min of incubation at 37°C, with slight agitation, 20 µL of the rFliC-WVDC-2109 mixture and 2.5 × 10^4^ HEK-Blue-hTLR5 cells were added in duplicate into flat-bottom 96-well plates. SEAP activity was measured at 655 nm using SpectraMax i3 spectrophotometer (Molecular Devices LLC) after 16 h of incubation at 37°C and 5% CO_2_.

### *In silico* monoclonal antibody docking analysis

The structure of WVDC-2109 was predicted using the DeepAb antibody structure prediction tool (36). The FliC structures for both *P. aeruginosa* and *B. pseudomallei* were predicted by AlphaFold (37, 38). The monoclonal antibodies and FliC protein structures were docked using the ClusPro protein-protein docking tool (39, 40). Antibody mode was used for docking prediction (41). The structures were visualized using UCSF ChimeraX (29, 42).

### Complement bactericidal assay

Complement-mediated bactericidal activity of WVDC-2109 mAb was determined with 30% (vol/vol) Guinea Pig Complement (MP Biomedicals, Fisher Scientific, #ICN642831), 30 µg/mL of WVDC-2109 or IgG2b Isotype Control (Bio X Cell #BE0086) and 10^6^ CFU/mL *P*. *aeruginosa* PAO1 or PAK grown to mid-log phase in LB, in a final volume of 100 µL. In some experiments, the guinea pig complement was heat-inactivated by treatment at 56°C for a minimum of 30 min. Samples were taken at time 0 and 90 min after incubation under gentle shaking at 37°C. To determine the viable bacteria at each time point, samples were serially diluted in PBS and plated on PIA. The percentage of bacterial survival was calculated using the number of bacteria at time 90 min relative to the number of bacteria at time 0 min.

### Macrophage opsonophagocytosis assay

Macrophage phagocytosis was determined using J774A.1 (ATCC: TIB-67) macrophages. Macrophages were maintained by culture in Dulbecco’s modified Eagle medium (DMEM) supplemented with 10% fetal bovine serum (FBS) and 1% penicillin/streptomycin (P/S). For the assay, approximately 1–2 × 10^6^ cells/mL macrophages were harvested and resuspended in minimal essential medium (MEM) supplemented with 1% BSA. Macrophages were then exposed at a multiplicity of infection (MOI) of 10:1 (bacteria:macrophages) to *P. aeruginosa* PAO1 (or PAK) grown to mid-log phase in LB and resuspended in MEM +1% bovine serum albumin (BSA). In some instances, 30 µg/mL WVDC-2109 or IgG2b Isotype Control (Bio X Cell #BE0086) were added to the suspension. MEM +1% BSA was used as a negative control. Suspensions were then incubated for 2 h with end-over-end rotation at 37°C. The number of viable bacteria was determined at time 0 and 2 h using serial dilutions in PBS and plating on PIA. The percentage of bacterial survival was calculated using number of bacteria at time 2 h relative to the number of bacteria at time 0.

For assessment of opsonophagocytosis in the presence of complement, an identical procedure was followed with the addition of 30% guinea pig Complement (MP Biomedicals, Fisher Scientific, #ICN642831) and incubation at 37°C for 30 min. Heat-inactivated guinea pig complement was used as a negative control. Macrophages were incubated at an MOI of 10:1 in the presence of 30 µg/mL WVDC-2109, IgG2b Isotype Control (Bio X Cell #BE0086), or MEM +1% BSA for 2 h using end-over-end rotation at 37°C. The amount of viable bacteria at time 0 and 2 h was determined by serial dilutions in PBS and plating on PIA. The percentage of bacterial survival was calculated using number of bacteria at time 2 h relative to the number of bacteria at time 0.

### Bacterial cell imaging and clumping analysis

A suspension of 10^6^ CFU/mL *P*. *aeruginosa* PAO1 grown to mid-log phase in LB was incubated with 30 µg/mL WVDC-2109, IgG2b Isotype Control, an anti-*P*. *aeruginosa* LPS antibody WVDC-0496 (43), or sterile PBS with end-over-end rotation at room temperature for 30 min. Following incubation, 30 µL of the samples were loaded into micro clear, flat bottom plates (Greiner Bio-One #655090), and heat-fixed in a drying oven set to 80°C for approximately 30 min until liquid has evaporated. Once samples were heat-fixed, a partial Gram stain was performed. Wells were flooded with 50 µL Crystal Violet (Fisher Scientific #270-180, 0.41% crystal violet wt/vol, 12% ethanol vol/vol, 0.1% phenol vol/vol, <90% deionized water vol/vol) for 1 min, and washed gently with deionized water for 10 seconds. Wells were then flooded with Gram’s Iodine mordant for 1 min and gently washed again. Wells were imaged using a Lionheart LX (Agilent BioTek) under 60× magnification on a bright field, with images obtained in a 2 × 2 grid and multiple regions of interest (ROI) obtained. Images were analyzed using Cell Profiler software and converted to a grayscale image to obtain cell size, count, and clustering parameters. Particles less than 1 micron square were filtered out, with the remainder being marked as “Bacteria.” The percentage of these cells that were found in clusters per image and the number of objects per image clusters was calculated.

### Mouse models of infection

*In vivo* efficacy of WVDC-2109 was determined in a prophylaxis model of sepsis and murine pneumonia. The lethal sepsis model was performed as previously described, with some modifications (32). Nine-week-old female outbred CD-1 mice were administered IP 45 mg/kg of WVDC-2109 diluted in PBS or an isotype IgG murine antibody as a negative control (BioXcell, #BE0086). After 12 h, mice were challenged IP with 5 × 10^6^ CFU of *P. aeruginosa* PAO1 from an exponential culture. Mice were monitored for 7 days to determine the survival rate. Mice were also scored for morbidity attending to six different criteria: (i) appearance, (ii) activity, (iii) eyes closure, (iv) respiration quality, (v) temperature loss, and (vi) body weight loss. Each variable was given a score from 0 (no symptoms) to 4 (worst symptoms). Mice were euthanized when they were moribund, which was determined as reaching a score of 4 in any of the variables or an accumulative score of 12 or above, following protocols approved by the West Virginia University Institutional Animal Care and Use Committee.

To measure bacterial burden in blood, spleen, and kidneys, mice were immunized and infected as described above. After 6 or 12 h of the challenge, mice were euthanized using 390 mg/kg of pentobarbital (Patterson Veterinary, #07-805-9296). Blood was collected via cardiac puncture. The blood was used to determine CFUs by serial dilution and plating as described previously and to obtain serum by centrifugation. The spleen and kidneys were removed under sterile conditions and homogenized. Bacteria present in these organs were quantified by diluting and plating the samples on PIA. Data were represented as the percentage of bacterial reduction in the WVDC-2109 compared to the isotype control group.

For the acute pneumonia model, 6-week-old female CD-1 mice were IP administered 45 mg/kg of WVDC-2109 or an isotype control antibody. As determined in previous studies performed in our lab (44), as a positive control, mice were passively immunized with serum from mice previously vaccinated with heat-inactivated *P. aeruginosa* PAO1 (*Pa-*WCV). To compare the protective activity of both monoclonal antibodies and the polyclonal serum used as positive control, the polyclonal serum was tittered against *P. aeruginosa* PAO1 by ELISA. Mice immunized with *Pa-*WCV sera received two times the equivalent titers compared with WVDC-2109 monoclonal antibody to guarantee a protective effect in those mice. After 12 h, mice were anesthetized with ketamine (77 mg/kg) (Patterson Veterinary, #07–803-6637) and xylazine (7.7 mg/kg) (Patterson Veterinary, #07-808-1939) in 0.9% saline and infected intranasally (IN) with 20 µL (2 × 10^7^ CFU) of *P. aeruginosa* PAO1. Approximately 14–16 h after infection, mice were euthanized using 390 mg/kg of pentobarbital. Blood was collected *via* cardiac puncture and serum was obtained by centrifugation and stored at −80°C until needed. The lungs were removed under sterile conditions and weighed. The right lobes were homogenized using GentleMACS dissociator (Miltenyi Biotec). Nasal washes were obtained by flushing 1 mL of PBS through the nasal cavity. Bacteria present in the lungs and nasal cavity were quantified by plating serial dilutions on PIA. All plates were incubated overnight at 37°C before counting CFUs.

To determine the pharmacokinetics of WVDC-2109 and compare it with *Pa*-WCV serum, mice received WVDC-2109 or *Pa*-WCV serum as described above. Mice were bled through the submandibular vein and serum was obtained after 1, 7, and 28 days. Antibody titers against *P. aeruginosa* PAO1 were determined by ELISA, as described above.

All animal experiments were performed according to the National Institutes of Health Guide for the Care and Use of Laboratory Animals. The animal protocol used in this study was approved by the West Virginia University Institutional Animal Care and Use Committee (WVU-ACUC protocol 1606003173).

### Histopathological analysis

The left lobes of the lung were embedded in 10% buffered formalin (VWR, #16004–128) for pathological examination. Fixed samples were embedded in paraffin and cut into 5 µm sections. Histology micrographs were obtained after staining with hematoxylin and eosin to assess for acute inflammation and with Gram stain to evaluate the presence of bacilli. The level of inflammation and presence of Gram-negative bacilli was scored by a certified pathologist.

### Cytokine measurements

CXCL-1, TNF-α, IL-1β, IL-6, IL-10, and IFN-γ levels present in serum (sepsis model) and lung homogenate supernatant (pneumonia model) were determined using a Mouse Magnetic Luminex Assay (R&D Systems, #LXSAMSM) following the manufacturer’s instructions.

### Statistical analysis

All data presented here were analyzed using the software Prism version 9.5.0 (GraphPad Prism). For more than three groups, comparisons were performed with a one-way analysis of variance (ANOVA). To compare the two groups, unpaired Student’s *t*-test (parametric) or Mann-Whitney test (non-parametric) were used. Differences in survival curves were assessed using a Log-rank Mantel-Cox test. Differences were considered statistically significant when *P* < 0.05.

## RESULTS

### Generation of broadly reactive mAbs against *P. aeruginosa* and *B. pseudomallei*

**Fig 1:**
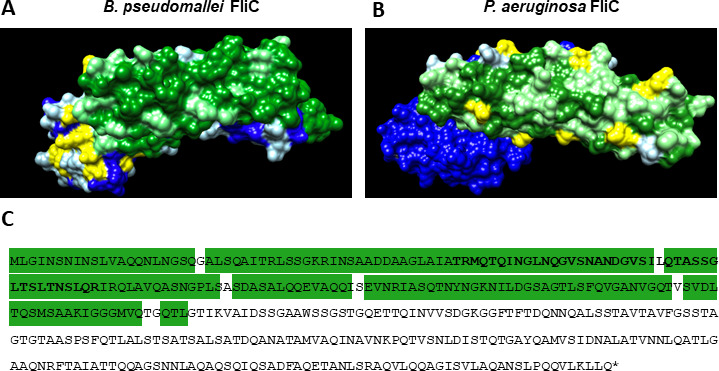
*B. pseudomallei* and *P. aeruginosa* flagellin variability map. 3D representation of *B. pseudomallei* (**A**) and *P. aeruginosa* (**B**) FliC proteins. Residues colored in dark green represent amino acids 100% conserved across all sequences compared, light green highlights residues with one polymorphism, and yellow and white residues with two or more polymorphisms. (**C**) Sequence of *B. pseudomallei* FliC with the peptide selected for immunization in bold letter. In addition, residues highly conserved across species (one polymorphism/position or less) are highlighted in green.

Previous studies have demonstrated that mAbs targeting surface-exposed components can be protective against infections caused by *Burkholderia* species as well as *P. aeruginosa* (45–47). We aimed to take advantage of this using a common antigenic sequence of flagellin to induce broadly reactive antibodies. A flagellin-derived peptide conserved among both pathogens was designed as an antigen to generate mAbs able to broadly recognize and bind *P. aeruginosa* and *B. pseudomallei*. The peptide was designed using multiple alignments across FliC sequences of *B. pseudomallei* and *P. aeruginosa*, as well as sequences from bacteria belonging to other Pseudomonadaceae and the families of *Vibrionaceae* and *Xanthomonadaceae*. Regions of high homology were then compared to known immunogenicity data for the protein FliC published in the literature (30, 31). This procedure led to the selection of a 40 amino acid peptide (Fig. 1). The peptide was conjugated to diphtheria toxoid CRM_197_. Based on previous studies, each CRM_197_ is expected to have 6–7 peptides conjugated onto the carrier (48). Mice were vaccinated with the FliC-CRM peptide and inguinal lymph nodes and splenocytes from the vaccinated mouse showing the highest antibody titer against *P. aeruginosa* and *B. pseudomallei* were extracted and fused with myeloma cells. The resulting hybridomas were cultured and subjected to several rounds of screenings and down-selection. ELISA was then used to select for hybridomas producing IgG against the FliC-derived peptide used for vaccination, recombinant *P. aeruginosa* FliC protein, and whole-cell *B. pseudomallei* Bp82 and *P. aeruginosa* PAO1. Of the total hybridomas produced, 5.1% met the criteria of being positive against the FliC-derived peptide and both pathogens *B. pseudomallei* and *P. aeruginosa* and were selected for further analysis. Among the hybridomas selected through our down-selection pipeline, a linear correlation was observed between the different antigens (Fig. S1). Hybridomas showing elevated absorbance readings against the recombinant FliC protein also demonstrated high binding against the FliC-derived peptide, whole-cell *B. pseudomallei,* and whole-cell *P. aeruginosa*. Among these antibody-producing hybridomas, a clone producing an IgG2bκ antibody (Fig. S2), herein referred to as WVDC-2109, was selected, purified, and subjected to further characterization.

**Fig 2:**
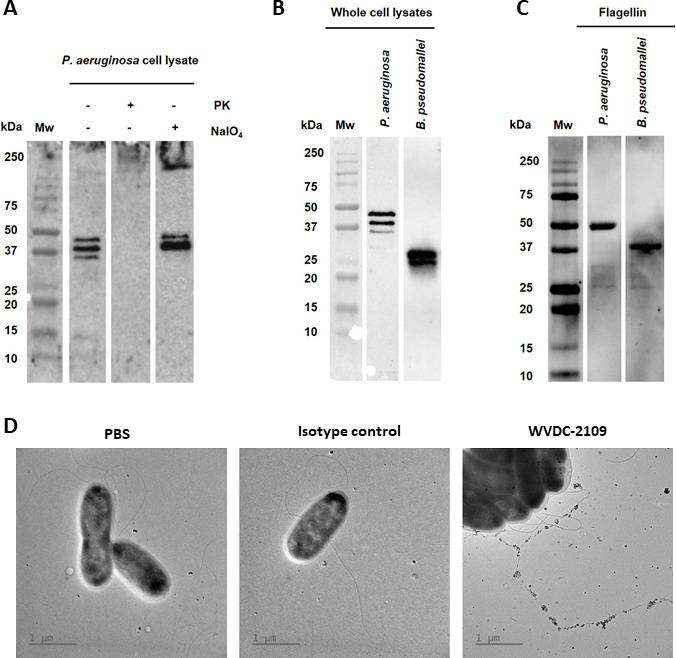
WVDC-2109 binds to *P. aeruginosa* and *B. pseudomallei* FliC. (**A**) Whole-cell lysates of *P. aeruginosa* PAO1 were treated with proteinase K (PK) or sodium periodate (NaIO_4_) and immunoblotted with WVDC-2109. (**B**) Immunoblot against 10 µg of *P. aeruginosa* PAO1 and *B. pseudomallei* Bp82 cell lysates using WVDC-2109 antibody. (**C**) Immunoblot using WVDC-2109 against 3 µg of recombinant FliC purified from *P. aeruginosa* or *B. pseudomallei* Bp82. (**D**) Transmission electron microscopy with immunogold labeling of *P. aeruginosa* using PBS, an isotype control or WVDC-2109 followed by a gold-conjugated secondary antibody. All blots in this panel were performed in parallel. Mw, Molecular weight size.

Various studies were then initiated to confirm the target of WVDC-2109. Western blotting was first used to determine the binding of WVDC-2109 to *P. aeruginosa* whole-cell lysate treated with proteinase K or sodium periodate (49). We observed that treatment of *P. aeruginosa* PAO1 lysates with proteinase K eliminated antigen recognition by WVDC-2109 while treatment with sodium periodate did not (Fig. 2A). Furthermore, analysis by western blotting of whole-cell lysate from *P. aeruginosa* and *B. pseudomallei* showed that WVDC-2109 was able to recognize both pathogens when resolved by SDS-PAGE (Fig. 2B). In native conditions, where flagellin is observed mostly as a multimer, WVDC-2109 also recognized whole-cell lysates as well as supernatants from several *P. aeruginosa* strains (Fig. S3). Western blotting analysis also confirmed WVDC-2109 binding to recombinant FliC from both pathogens (Fig. 2C). To further determine WVDC-2109 binding to the bacterial flagella, immunogold-labeling and transmission electron microscopy were used. In this assay, WVDC-2109 bound to *P. aeruginosa* flagella (Fig. 2D) while no binding was observed when the isotype control was used. Altogether, these data support that WVDC-2109 recognizes and binds to *P. aeruginosa* PAO1 and *B. pseudomallei* Bp82 flagella.

### WVDC-2109 interacts with flagellin without blocking TLR5 activation

**Fig 3:**
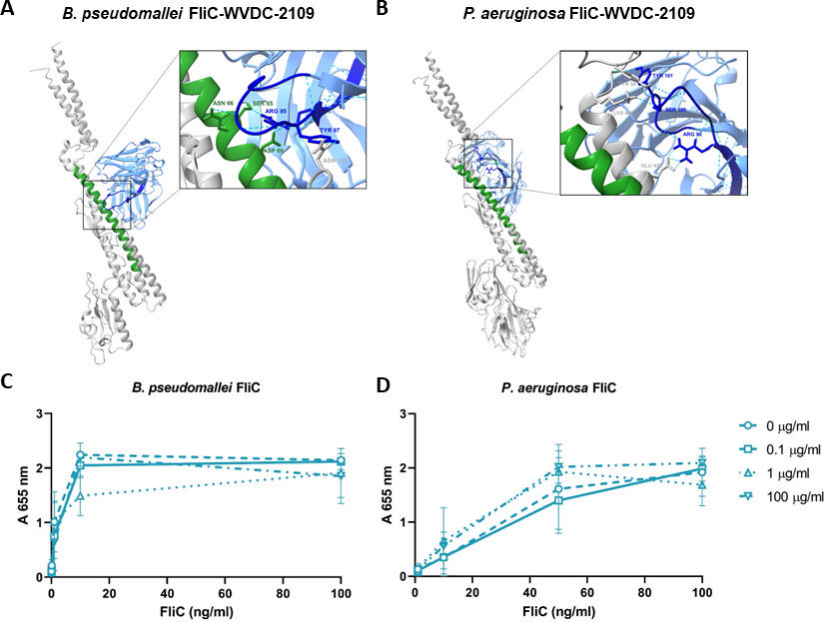
WVDC-2109 interacts with FliC without blocking TLR5 activation. Model of the interaction between *B. pseudomallei* FliC (**A**) and *P. aeruginosa* FliC (**B**) and the predicted structure of WVDC-2109. In both panels A and B, the structure of FliC is depicted in gray. The WVDC-2109 antibody is illustrated in light blue, with residues unique to the WVDC-2109 heavy chain highlighted in dark blue. The peptide sequence is shown in green, indicating the sequence and its corresponding similar peptide in *P. aeruginosa* used for generating hybridomas. The amino acid residues in the heavy chain variable region involved in binding to FliC are shown in a box. (**C**) HEK-Blue hTLR5 cells stimulated with *B. pseudomallei* flagellin (0–100 ng/mL) were previously incubated with increasing concentrations of WVDC-2109 (0–100 µg/mL). (**D**) HEK-Blue hTLR5 cells stimulated with *P. aeruginosa* flagellin (0–100 ng/mL) were previously incubated with increasing concentrations of WVDC-2109 (0–100 µg/mL). SEAP activity was determined with HEK-Blue detection media at 655 nm. Bars graphs represent mean ± SD.

During bacterial infection, flagellin produced by bacterial pathogens is able to bind Toll-like receptor (TLR) 5 and trigger TLR5-mediated signaling (50). It has been previously demonstrated that, unlike other TLRs such as TLR3 and TLR9, TLR5-mediated immune stimulation does not increase the risk of exacerbated inflammation and tissue destruction (51). On the contrary, host inflammatory response *via* TLR5 seems to be protective in *P. aeruginosa* infections (52, 53). Using computational tools, the binding of WVDC-2109 with *P. aeruginosa* and *B. pseudomallei* FliC was predicted (Fig. 3A and B). The models suggest that WVDC-2109 can bind to both *B. pseudomallei* FliC and *P. aeruginosa* FliC. In *B. pseudomallei*, the amino acid residues in the FliC peptide used for hybridoma generation and immunization are predicted to be involved in the interaction with WVDC-2109 with FliC. However, this interaction was not observed in *P. aeruginosa* FliC and its corresponding FliC peptide (Fig. 3A and B).

Previous studies have shown that amino acids involved in FliC-TLR5 interaction are highly conserved among flagellated Gram-negative pathogens (54, 55). To test whether the presence of WVDC-2109 antibody interferes with TLR5 activation, purified recombinant FliC from *B. pseudomallei* and *P. aeruginosa* were pre-incubated with WVDC-2109 for 90 min and then added the mixture to human HEK-Blue-hTLR5 cells. In this assay, flagellin from both pathogens was immunostimulatory and triggered TLR5 signaling, even at low concentrations of flagellin (10 ng/mL). The presence of the antibody did not block the activation of the cells mediated by FliC at the concentrations tested (Fig. 3C and D). Taken together, these results suggest that WVDC-2109 binds to FliC from *B. pseudomallei* and *P. aeruginosa* without blocking TLR5 activation.

### WVDC-2109 increases complement-mediated and opsonophagocytic killing of *P. aeruginosa*

Monoclonal antibodies can induce bacterial clearance *via* aggregation, activating complement-mediated lysis, or opsonophagocytosis mediated by macrophages. To first determine the effect of WVDC-2109 on bacterial aggregation, *P. aeruginosa* PAO1 was incubated either in the presence of WVDC-2109, an isotype control as negative control, and WVDC-0496, and anti-*P*. *aeruginosa* LPS antibody that promotes bacterial aggregation as a positive control (43). Imaging of the bacterial suspensions following incubation indicated that only the anti-LPS antibody was able to generate bacterial aggregation, as only this antibody led to the statistically significant formation of clusters compared to bacteria alone (Fig. S4).

**Fig 4:**
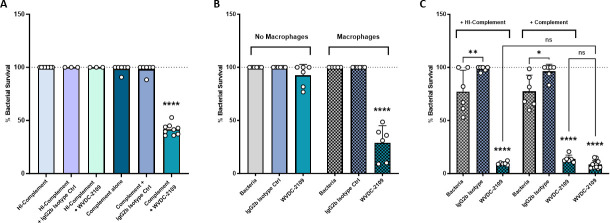
WVDC-2109 increases bacterial killing mediated by the complement system and opsonophagocytic killing by macrophages. (**A**) Complement bactericidal assay. Percentage of survival of *P. aeruginosa* PAO1 after 90 min of incubation with Heat-Inactivated (HI) Guinea Pig Complement, Guinea Pig Complement alone, HI-Guinea Pig Complement + IgG2 b Isotype control, HI-Guinea Pig Complement + WVDC-2109, Guinea Pig Complement + IgG2 b Isotype control, or Guinea Pig Complement + WVDC-2109. **** Denotes a comparison to all other groups. (**B**) Opsonophagocytic killing assay. Percentage of survival of *P. aeruginosa* PAO1 after 2 h of incubation with macrophage J774A.1 cells previously opsonized with or without WVDC-2109 or IgG2b isotype control. **** Denotes a comparison to all other groups. (**C**) Opsonophagocytic killing assay with the addition of HI-Guinea Pig Complement or Guinea Pig Complement. Percentage of survival of *P. aeruginosa* PAO1 after 2 h of incubation with macrophage J774A.1 cells previously opsonized with or without WVDC-2109 or IgG2b isotype control, with or without added HI-Guinea Pig Complement or Guinea Pig Complement. **** Denotes a comparison to all other groups, unless otherwise displayed. Statistical significance was determined by ordinary one-way ANOVA: *P < 0.05, **P < 0.01, ****P < 0.0001. The dotted line represents the above growth threshold. Each dot represents one replicate. Error bars represent the standard error of the mean.

Given the importance of the complement system, many pathogens have developed strategies to evade it, including *P. aeruginosa* and *B. pseudomallei* (56, 57). To determine whether the presence of WVDC-2109 could increase *P. aeruginosa* PAO1 complement-dependent killing, bacteria were incubated with guinea pig complement alone or with WVDC-2109 (Fig. 4A). Heat-inactivated complement and an isotype control were used as negative controls. In these studies, complement alone was not sufficient to significantly decrease bacterial survival. However, the addition of WVDC-2109 to the complement source significantly decreased bacterial viability by 71.09% compared to complement alone (Fig. 4A), despite the intrinsic resistance of *P. aeruginosa* to complement-mediated killing (56, 58). This was not observed when *P. aeruginosa* PAO1 was incubated with WVDC-2109 and heat-inactivated complement. In addition, this effect was specific to WVDC-2109 as incubation of *P. aeruginosa* with complement and an isotype control antibody did not lead to changes in bacterial viability (Fig. 4A).

To evaluate whether WVDC-2109 could also facilitate bacterial uptake by phagocytic cells, the ability of J774A.1 macrophages to kill *P. aeruginosa* PAO1 previously opsonized with WVDC-2109 or an isotype control antibody was determined. In this assay, WVDC-2109 did not increase the attachment of the bacteria to the macrophages (Fig. S5A). Moreover, the antibody alone was not sufficient to reduce bacterial viability (Fig. S5B). However, the opsonization of *P. aeruginosa* with WVDC-2109 decreased the amount of viable bacteria exposed to J774A.1 macrophages compared to macrophages alone (Fig. 4B). This effect was specific to WVDC-2109 and was not observed with the use of an isotype control antibody.

Finally, to determine whether the presence of complement can enhance macrophage opsonophagocytic killing, *P. aeruginosa* PAO1 was first opsonized with WVDC-2109 or isotype control and either heat-inactivated or active guinea pig complement, prior to exposure to J774A.1 macrophages. In this assay, the viability of *P. aeruginosa* was significantly reduced by the presence of WVDC-2109 as observed in Fig. 4B. This was specific to WVDC-2109 as treatment with the isotype control did not affect bacterial viability. In addition, the addition of complement did not further reduce bacterial viability, suggesting that in this specific context, complement does not enhance macrophage opsonophagocytic killing (Fig. 4C). In the absence of J774A.1 macrophages, we observed no decrease in *P. aeruginosa* bacterial viability with the exception of WVDC-2109 with added active guinea pig complement (Fig. S6A). To evaluate the effect WVDC-2109 has against other types of FliC *Pseudomonas* strains, we analyzed the ability of both the complement system and J774A.1 macrophages against *P. aeruginosa* PAK, a representative strain that possesses a Type A flagellin. We observed an overall lack of bacterial killing, for both the complement system and opsonophagocytosis, for WVDC-2109 against the PAK strain (Fig. S6B and C). These results show that the opsonophagocytic ability of WVDC-2109 against *P. aeruginosa* PAO1 is not altered by the presence of complement.

### WVDC-2109 protects against *P. aeruginosa* lethal bacteremia

**Fig 5:**
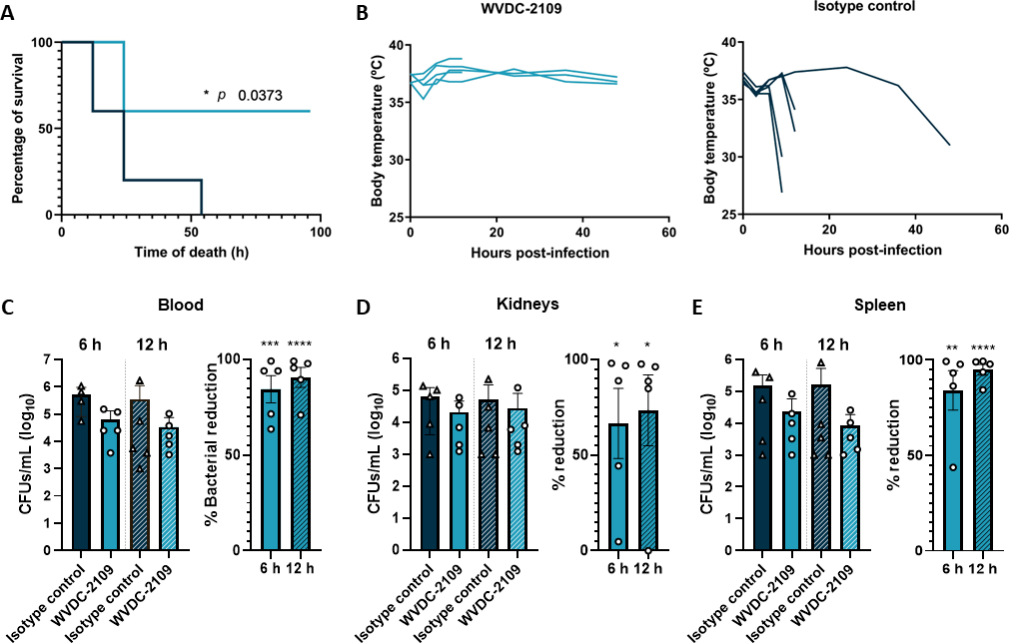
WVDC-2109 protects mice against *P. aeruginosa* sepsis. Mice survival (**A**) and rectal temperatures (**B**) over time of mice challenged with *P. aeruginosa* in a murine lethal model of sepsis. Mice received WVDC-2109 (light blue) or an isotype IgG control (dark blue) 12 h before *P. aeruginosa* PAO1 IP infection and were monitored for morbidity and mortality over 96 h. Bacterial burden in the blood (**C**), kidneys, (**D**) and spleen (**E**) 6 and 12 h after *P. aeruginosa* PAO1 IP infection in mice that were prophylactically administered WVDC-2109 or an isotype control antibody. Statistical significance in A was determined using a Mantel–Cox test. Differences in bacterial burden were determined using the Mann-Whitney test. *P < 0.05.

Bloodstream infections (BSI) caused by *P. aeruginosa* represent 8.5% of all BSIs, with the highest mortality rate among causative agents (59). To investigate the impact of the WVDC-2109 in *P. aeruginosa* BSI, the capacity of the antibody to improve survival was tested in a lethal mouse model of sepsis. Mice received WVDC-2109 or a non-specific IgG2b as control and were challenged IP 6 h later with a lethal dose of *P. aeruginosa* PAO1. Morbidity was monitored for the subsequent 5 days. While all control mice that received a passive immunization with the isotype control antibody became morbid and displayed a dramatic temperature loss within the first 60 h after challenge, WVDC-2109 prophylactic treatment significantly increased survival of mice by 60% (Fig. 5A). Mouse survival was associated with the maintenance of stable body temperature over time in WVDC-2109-treated mice, while the body temperature of 4 out of 5 isotype-treated mice dropped below 35°C post-challenge (Fig. 5B). To determine whether antibody administration was associated with changes in bacterial burden and dissemination, bacterial loads in the blood, kidney, and spleen were determined 6 and 12 h post-challenge. Although non-statistically significant, a reduction of viable bacteria by 87% and 90% was observed in the blood (Fig. 5C), 66% and 73% in the kidneys (Fig. 5D), and 84% and 94% in the spleen (Fig. 5E) at 6 and 12 h, respectively, after *P. aeruginosa* infection in mice treated with WVDC-2109 compared with mice passively immunized with a non-specific isotype control antibody. Moreover, while non-statistically significant, cytokine levels (TNFα, IL-1β, IL-6, IL-10, and IFNɣ) were also lower in the serum of mice that received WVDC-2109 (Fig. S7), suggesting lower levels of inflammation in antibody-treated mice. These data together illustrate that prophylaxis with WVDC-2109 protects against *P. aeruginosa* bloodstream infection by preventing death in a lethal sepsis model.

### WVDC-2109 reduces bacterial burden and edema in the respiratory tract during murine *P. aeruginosa* acute pneumonia

**Fig 6:**
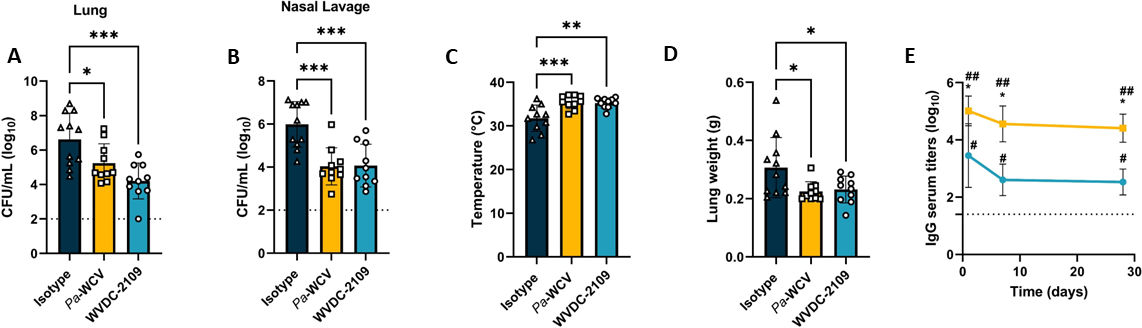
WVDC-2109 reduces bacterial burden and lung edema during acute murine pneumonia. Bacterial burden in the respiratory tract 16 h after *P. aeruginosa* challenge in CD-1 mice. Mice received WVDC-2109, a non-specific isotype IgG control or *Pa-*WCV serum 12 h before *P. aeruginosa* PAO1 infection. Viable bacteria were quantified in the lung (**A**) and nasal lavage (**B**) using serial dilutions. Rectal temperature (**C**) and lung mass (**D**) were also determined at the time of euthanasia. (**E**) Pharmacokinetics of WVDC-2109. Mice were injected with IP WVDC-2109 (blue) or *Pa*-WCV serum (orange). The level of anti-*P*. *aeruginosa* PAO1 IgG in the mice sera was determined by ELISA. Each dot represents an individual animal. Results from statistical analysis of comparisons between animals treated with *Pa*-WCV serum and animals administered WVDC-2109 are represented with *, and *Pa*-WCV and WVDC-2109 to the limit of detection are represented with #. Statistical significance was determined by one-way ANOVA in A-D (*P < 0.05, **P < 0.005, ***P < 0.0005), and one-sample *t*-test and Mann-Whitney test in E (^#^*P* < 0.05, ^##^*P* < 0.01; *P < 0.05). The dotted line indicates the lowest limit of detection.

The efficacy of WVDC-2109 was further evaluated in another clinically relevant *in vivo* model of *P. aeruginosa* infection: a murine model of acute pneumonia. Passive immunization was performed in CD-1 mice with WVDC-2109 or a non-specific isotype control antibody. Previous studies performed in our laboratory have demonstrated that a whole-cell heat-inactivated *P. aeruginosa* (WCV) vaccine is protective in mice and that passive immunization of naïve mice using serum from WCV mice protected them against *P. aeruginosa* acute pneumonia (44). So, as a positive control for protection, we used mice passively immunized with WCV serum (*Pa*-WCV). Twelve hours after injection, all mice were challenged IN with *P. aeruginosa* PAO1. Fourteen to sixteen hours post-challenge, mice were euthanized, and rectal temperature, wet lung mass, and bacterial burden in the respiratory tract were evaluated. Similar to the results seen in the sepsis model, while bacterial loads in the lung and the nares of mice immunized with the isotype control were high, treatment with WVDC-2109 significantly reduced bacterial burden in the lung and in the nares by 99% and 98%, respectively, compared to the isotype control-treated mice (Fig. 6A and B). Moreover, mice administered WVDC-2109 prophylactically were also able to maintain body temperature and lung wet mass similar to non-challenged mice, which suggests reduced pulmonary edema in those animals (Fig. 6C and D) Interestingly, the decrease in bacterial burden in the lung was more pronounced in mice passively immunized with WVDC-2109 than mice that received *Pa*-WCV serum (96% of decrease, compared to the isotype control). This reduction was observed despite the fact that significantly higher levels of antibodies against *P. aeruginosa* were detected in mice that had received *Pa-*WCV serum compared to WVDC-2109 (Fig. 6E).

**Fig 7:**
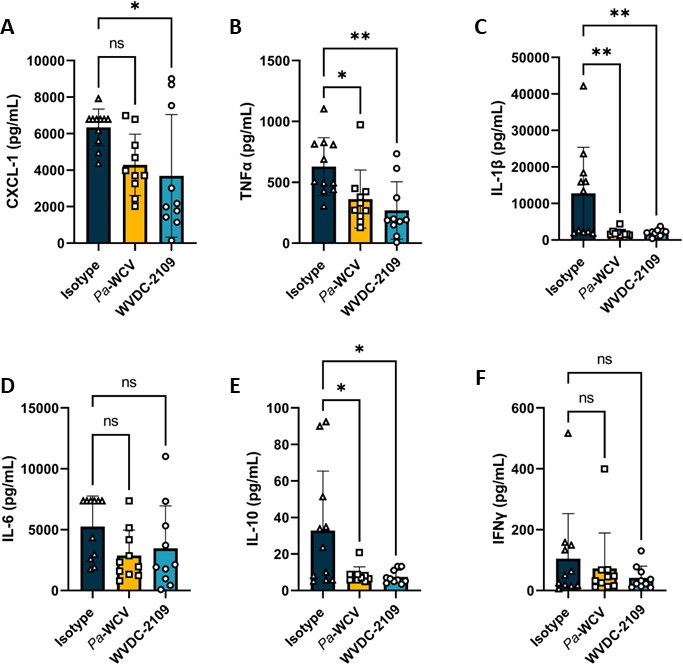
WVDC-2109 reduces lung inflammation during acute murine pneumonia. CXCL-1 (**A**), TNFα (**B**), IL-1β (**C**), IL-6 (**D**), IL-10, (**E**) and IFN-γ (**F**) in the lung homogenate of CD-1 mice after 16 h of *P. aeruginosa* PAO1 intranasal infection. Mice were passively immunized with WVDC-2109, with a non-specific isotype IgG as a negative control or serum from mice previously vaccinated with inactivated *P. aeruginosa* (*Pa* WCV) 12 h before *P. aeruginosa* PAO1 infection. Each dot represents an individual animal. Statistical significance was determined by one-way ANOVA. *P < 0.05, **P < 0.005. The dotted line indicates the lowest limit of detection.

To further determine the effect of WVDC-2109 on pulmonary inflammation, cytokines from the supernatant generated from lung homogenate were determined. Levels of CXCL-1/KC, TNFα, IL-1β, and IL-10 were significantly lower in the lung of mice previously immunized with WVDC-2109 compared with mice that received an isotype control antibody (Fig. 7A, B, C, and E). Levels of IL-6 and IFNγ were also lower in mice treated with WVDC-2109, although non-statistically significant (Fig. 7D and F).

**Fig 8:**
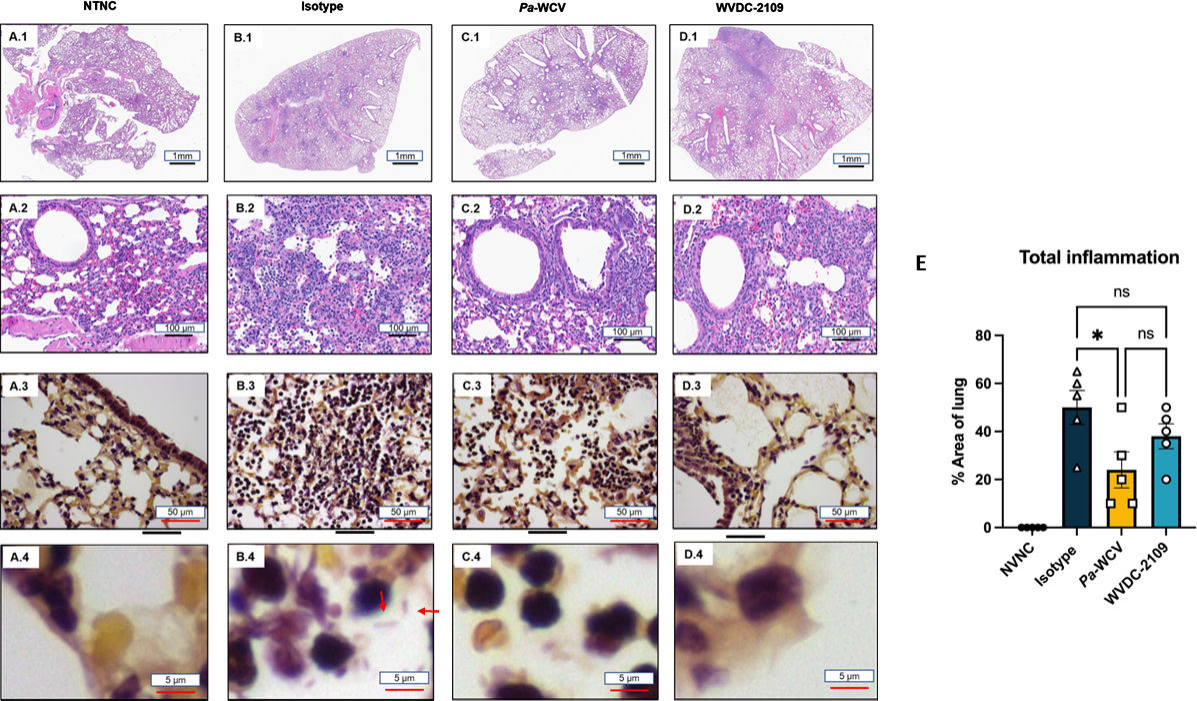
Representative hematoxylin and eosin and Gram-stained sections obtained from the right lobe of the murine lungs. (**A**) Non-treated non-challenged (NTNC) mouse lung depicting (**A.1**) unremarkable pulmonary parenchyma and vasculature without evidence of inflammation (1×), (**A.2**) typical lung architecture consisting of alveolar spaces, bronchiole, and vessels with absence of inflammatory cells (20×) and (A.3, A.4) Gram stain revealing no bacilli in alveolar spaces, interstitium, or bronchial lumen (60×). (**B**) Isotype control mouse shows widespread acute inflammation (1×) (B.1), acute inflammation characterized by neutrophilic invasion of alveolar spaces and interstitium (20×). (**B.3**) Gram stain shows viable Gram-negative rods in alveolar spaces in a background of diffuse neutrophilic infiltration (60×). (**C**) *Pa*-WCV serum group mouse lung showing decreased net inflammation largely localized to peribronchial zones (1×) (C.1), a pair of bronchioles circumscribed by neutrophils characteristic of peribronchial inflammation (20×). (**C.3, C.4**) Gram stain depicting neutrophils infiltrating alveolar spaces without evidence of viable rods (60×). (**D**) WVDC-2109 inoculated mouse lung showing diffuse, acute inflammation (D.1) wish sheets of neutrophils infiltrating the alveoli (20×) (D.2). (D.3, D.4) Gram stain depicting scattered neutrophils with absence of viable bacilli (60×). (**E**) Percentage of the lung area affected by inflammation across all groups. Each dot represents an individual animal. Differences were determined using a one-way ANOVA with Dunnett´s multiple comparison test. *P < 0.05.

These results were confirmed with the histology micrographs obtained from the right lobe of the infected mice (Fig. 8A through D). We observed that the mean alveolar area where inflammation was observed was lower in animals that received WVDC-2109 (Fig. 8D) compared with the isotype control (Fig. 8B), although the percentage of reduction was non-statistically significant (Fig. 8E). Treatment with *Pa*-WCV (Fig. 8C and E) was also able to reduce inflammation. Moreover, no viable bacilli were detected in mice treated with WVDC-2109 (Fig. 8D.3 and D.4) or the *Pa*-WCV serum (Fig. 8B.3 and B.4), while diffusely distributed bacilli were observed in the alveoli of the lung from mice isotype control group (Fig. 8C.3 and C.4). Overall, these data show that prophylaxis with WVDC-2109 can reduce bacterial colonization, edema, and inflammation in the lower respiratory tract during *P. aeruginosa* acute murine pneumonia.

### WVDC-2109 binds to *P. aeruginosa* clinical isolates, Burkholderia species, and other pathogens of concern

**Fig 9:**
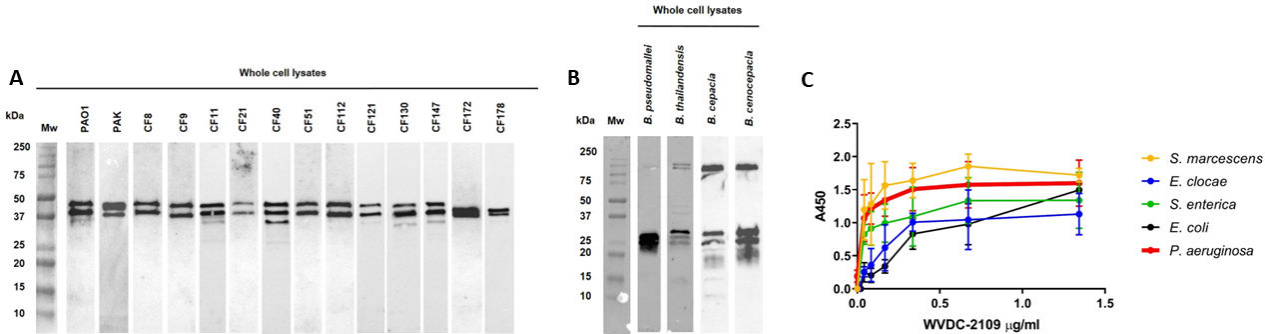
Cross-reactivity of WVDC-2109. (**A**) Western Blot using WVDC-2109 against 10 µg of WCL from *P. aeruginosa* PAO1, *P. aeruginosa* PAK and *P. aeruginosa* clinical isolates (CF8, CF9, CF11, CF21, CF40, CF51, CF112, CF121, CF130, CF147, CF172, and CF178). (**B**) Western blot against 10 µg of *B. pseudomallei, B. thailandensis, B. cepacia,* and *B. cenocepacia* WCL using WVDC-2109. All blots in this panel were performed in parallel. (**C**) ELISA using WVDC-2109 against whole *S. marcescens, E. clocae, S. enterica, E. coli*, and *P. aeruginosa*.

*Pseudomonas aeruginosa* is in part characterized by a high genetic diversity among different isolates, especially in chronic infections, which represents a challenge for vaccine development (60). For example, *P. aeruginosa* expresses two different types of flagellin, type a flagellin, and type b flagellin, with different molecular sizes. Each *P. aeruginosa* strain produces a single type of flagellin, and no switching between serotypes has been reported for any *P. aeruginosa* strain (61). For instance, the laboratory strains PAO1 and PAK express type b and type a flagellin, respectively. Western blot analysis demonstrated that WVDC-2109 was able to recognize both flagellin types (Fig. 9A). However, WVDC-2109 did not mediate opsonophagocytic killing of the strain PAK which expresses type a flagellin (Fig. S6B.6C). In addition, there is an association between the LPS O-serotype and flagellin type in *P. aeruginosa* (62). To determine whether WVDC-2109 was capable of binding different clinical isolates expressing different LPS serotypes, non-clonal and phenotypically diverse *P. aeruginosa* strains isolated from CF patients (Table S1) were selected. Western blotting showed that WVDC-2109 can bind to clinical isolates from all LPS serotypes tested in this study (O1, O3, O4, O5, O6, O9, and O11) (Fig. 9A).

As shown in Fig. 2, WVDC-2109 was also able to recognize *B. pseudomallei*. Given that flagellin is broadly conserved across *Burkholderia* species, the binding of WVDC-2109 to other *Burkholderia* species of interest was tested. Western blotting showed that WVDC-2109 recognized *B. thailandensis, B. cepacia,* and *B. cenocepacia* (Fig. 9B). Finally, the ability of WVDC-2109 to bind to other pathogens of concern was determined by ELISA. WVDC-2109 bound to the flagellated pathogens *Serratia marcescens, Enterobacter clocae, Salmonella enterica,* and *Escherichia coli* (Fig. 9C). Based on a preliminary IC50 analysis, the IC50 was lowest for *S. marcescens* (0.022 µg/mL), followed by *P. aeruginosa* (0.043 µg/mL), *E. cloacae* (0.138 µg/mL), and *E. coli* (3.985 µg/mL). Overall, binding analysis by western blot and ELISA showed that WVDC-2109 is a broad-spectrum antibody able to recognize a wide range of Gram-negative flagellated pathogens of concern.

## DISCUSSION

In this proof-of-concept study, we used a flagellin-derived peptide as an immunogen to generate antibodies against these bacterial species. We aimed to investigate whether peptides composed of conserved epitopes of numerous Gram-negative bacteria could serve as immunogens for the development of cross-reactive antibodies against flagellated microorganisms. Using the hybridoma technique, we successfully generated a cross-reactive antibody, WVDC-2109, which recognized several flagellated pathogens of interest, including *P. aeruginosa*, *Burkholderia* species, and other AMR pathogens (59). In the present study, we demonstrated that prophylactic administration of WVDC-2109 was protective in a lethal model of *P. aeruginosa* bacteremia and a model of acute pneumonia in mice. Protection was associated with a decrease in bacterial burden in blood, kidneys, and spleens (sepsis model), or in the nares and the lung (pneumonia model) of mice treated with WVDC-2109 compared with mice treated with an isotype control antibody. *In vitro* results suggest that the bacterial clearance *in vivo* could be attributed to the ability of WVDC-2109 to facilitate complement-mediated and opsonophagocytic killing. This finding holds significant importance as previous studies have established that complement is the main defensive mechanism against *P. aeruginosa* in human blood (63) and plays a crucial role in the initial stages of the infection (64–66) (67–73). In addition to complement, opsonophagocytic killing by neutrophils and circulating macrophages also plays an important role in the ability of the immune system to control *P. aeruginosa* infections (63, 74), as the bacteria is highly susceptible to phagocytosis mediated by the interaction between opsonic antibodies and phagocyte surface Fc receptors (75). In the absence of a complement system, WVDC-2109 significantly increased phagocytic killing mediated by macrophages suggesting that WVDC-2109 could contribute to the clearance of *P. aeruginosa in vivo* through this mechanism. Future studies should aim to evaluate the role of WVDC-2109 in opsonophagocytic killing by other innate immune cells such as neutrophils, and their relative contribution to clearance during infection

During infection, recognition of pathogen-associated molecular patterns (PAMPs) *via* pattern recognition receptors leads to the production of pro-inflammatory cytokines. While essential to initiate the innate immune response to infection, excessive pro-inflammatory cytokine responses can lead to inflammation-mediated tissue destruction, decline in lung function, and worsened patient outcomes (76, 77). Flagellin is a known PAMP that can bind to and trigger TLR5 signaling. During acute *P. aeruginosa* infection in mice, TLR5 signaling is associated with the clearance of low amounts of bacteria and the recruitment of neutrophils to the site of infection (78). In this study, we hypothesized that the binding of WVDC-2109 to flagellin would interfere with the binding of flagellin to TLR5, and hinder TLR5-mediated clearance. Interestingly, we observed that the binding of WVDC-2109 with recombinant flagellin from *P. aeruginosa* and *B. pseudomallei* did not inhibit TLR5 signaling in reporter cell lines *in vitro*. This result is likely due to the distance between the residues on flagellin known to interact with TLR5 and the binding region of the antibody (Fig. 3). This observation suggests that WVDC-2109 can provide protection *via* complement-mediated and opsonophagocytic killing without hindering the TLR5 pro-inflammatory response involved in the clearance of *P. aeruginosa*. In addition, the administration of WVDC-2109 as a prophylactic treatment in mice was associated with lower levels of pro-inflammatory cytokines including CXCL1/KC, IL-6, IFNγ, TNFα, and IL-1β (Fig. 7). Since administration of WVDC-2109 correlates with an increase in bacterial clearance, we speculate that the lower levels of circulating cytokines in response to infection present in mice treated with WVDC-2109 are associated with a greater control of bacterial burden rather than interference with TLR5 signaling. This finding holds special significance, as it suggests that WVDC-2109 treatment has the potential to attenuate the cytokine storm associated with severe *P. aeruginosa* infections and sepsis.

One caveat in the design of this approach is that not all *P. aeruginosa* strains and not all *Burkholderia* species are motile. In the case of *P. aeruginosa* infections, motility is often lost during chronic respiratory infections in patients with underlying diseases such as cystic fibrosis (18). In addition, not all *P. aeruginosa* strains produce the same type of flagellin (61). In this study, we demonstrated that WVDC-2109 binds to representative strains of *P. aeruginosa* expressing both type a and type b flagellin, and to all the clinical isolates of *P. aeruginosa* tested, regardless of their LPS serotype or *in vitro* motility (Fig. 9). Interestingly, WVDC-2109 opsonophagocytic killing properties were only observed in the presence of the type b flagellin expressing strain PAO1 and not the type b flagellin expressing strain PAK. It is unclear why despite binding to strains with both type a and b flagellin differences were observed in the levels of opsonophagocytic killing. Possible explanations include inherent differences in strain susceptibility to opsonophagocytic killing since the strains were not isogenic, varying levels of flagellin expression in each strain, or differences in antibody-binding affinity to each type of flagellin. In addition, we observed that WVDC-2109 also binds to bacterial species members of the ESKAPEE family of AMR-resistant pathogens. While further studies are necessary to determine the exact epitope recognized by WVDC-2109 in *E. coli, S. enterica*, *E cloacae*, and *S. marcescens*. These studies should also aim at determining the efficacy of WVDC-2109 against *Burkholderia* species and ESKAPEE pathogens *in vivo*.

These outcomes from this study underscore the advantages of immunization strategies employing peptides targeting conserved protein regions. Notably, a similar approach was used by Li et al. in their aim to develop a universal SARS-CoV2 vaccine (79). Using an S2-based peptide shared across SARS-CoV and various SARS-CoV2 strains, they achieved robust protection against diverse variants in mice (79). It is also worth highlighting that broadly neutralizing monoclonal antibodies, targeting linear peptides, have been isolated from HIV-infected patients (80). This finding prompted the development of immunogens capable of eliciting antibodies with the analogous broad spectrum to those natural antibodies. While this endeavor used a different technique in which the peptides containing the epitopes were transferred onto scaffold proteins (81), it underscores the advantages of peptide-based strategies in achieving a wide spectrum of activity. In the context of this study, this holds particular significance for treating lung bacterial infections in cystic fibrosis patients. Considering these infections, the co-isolation of *P. aeruginosa* and *B. cenocepacia* is usually associated with more severe infections (82).

Previous attempts to develop monoclonal antibodies against *P. aeruginosa* have led to efficacy testing in clinical trials (15, 83). Despite good safety profiles and promising results, none of them have progressed past phase III clinical trials and are available for use in the clinic. One possible explanation for the shortcomings of these antibodies in clinical trials is that they were administered for the treatment of *P. aeruginosa* infection. The data from this study suggest that antibodies against *P. aeruginosa* can be used for the prevention of infections caused by this organism. Such use is conceivable in patients admitted to the hospital and placed under assisted ventilation, or patients at risk of contracting *P. aeruginosa* infections admitted in the ICU. Another possible explanation for the failure of anti-*P*. *aeruginosa* antibodies in clinical trials is that they were administered to mechanically ventilated patients or cystic fibrosis patients, both of which often develop polymicrobial pneumonia (84, 85). Given the specificity of the monoclonal antibodies tested in clinical trials, achieving significant success in those types of infection can be challenging. We speculate that the use of WVDC-2109 could overcome this problem as it binds to several pathogens responsible for causing polymicrobial infections in cystic fibrosis patients, as well as various other AMR pathogens. A similar approach has been used to generate CMTX-001, a monoclonal antibody developed by Clarametyx Biosciences and currently in phase 1a of clinical trials (NCT05629741) (86). CMTX-001 recognizes the conserved regions of DNABII binding proteins, being able then to target polymicrobial biofilms. Another example of a cross-species protective antigen that could target several AMR pathogens is Poly-ß-(1-6)-^N^-Acetyl Glucosamine (PNAG), a surface polymer expressed by many AMR bacteria and an important component of biofilms. Antibodies against PNAG exhibit broad-spectrum efficacy against several pathogens such as *Staphylococcus aureus, Escherichia coli,* and Group B *Streptococcus* (87, 88). Therefore, a human IgG1 mAb directed against a deacylated form of PNAG, F598, displayed protective efficacy in mice models infected with different AMR bacteria (89). Notably, this mAb has progressed through phase 1 and 2 clinical trials (87, 88). Thus, it is likely that targeting several pathogens with one single broad-spectrum monoclonal antibody or a cocktail of multiple antibodies is necessary for the treatment of AMR infections in the clinic.

Overall, the data presented here describe a new broadly reactive monoclonal antibody able to recognize different pathogens of concern. We have demonstrated that this antibody has *in vitro* and *in vivo* efficacy against the pathogen *P. aeruginosa*. WVDC-2109 increased survival and reduced bacterial viability in a sepsis model of infection and reduced bacterial burden and overall lung inflammation in an acute model of pneumonia. Future efforts will focus on analyzing the pharmacokinetics and safety profile of this antibody, generating humanized clones, and determining their biological activity against additional pathogens of interest.

## SUPPLEMENTAL MATERIAL

Supplemental figures iai.00330-24-s0001.pdf Fig. S1 to S7.

Supplemental material iai.00330-24-s0002.docx Supplemental methods for Fig. S5.

Supplemental data iai.00330-24-s0003.xlsx Gram Negative Flux results.

Table S1 iai.00330-24-s0004.docx Bacterial strains used in the study.

ASM does not own the copyrights to Supplemental Material that may be linked to, or accessed through, an article. The authors have granted ASM a non-exclusive, world-wide license to publish the Supplemental Material files. Please contact the corresponding author directly for reuse.
