# Supplementary material for: Novel broadly reactive monoclonal antibody protects against Pseudomonas aeruginosa infection
Source: Infect Immun. 2024 Dec 13;93(1):e00330-24. doi: 10.1128/iai.00330-24 (PMC11784295; doi:10.1128/iai.00330-24)
Supplement: Supplemental figures — Fig. S1 to S7. [file iai.00330-24-s0001.pdf]

Figure S1

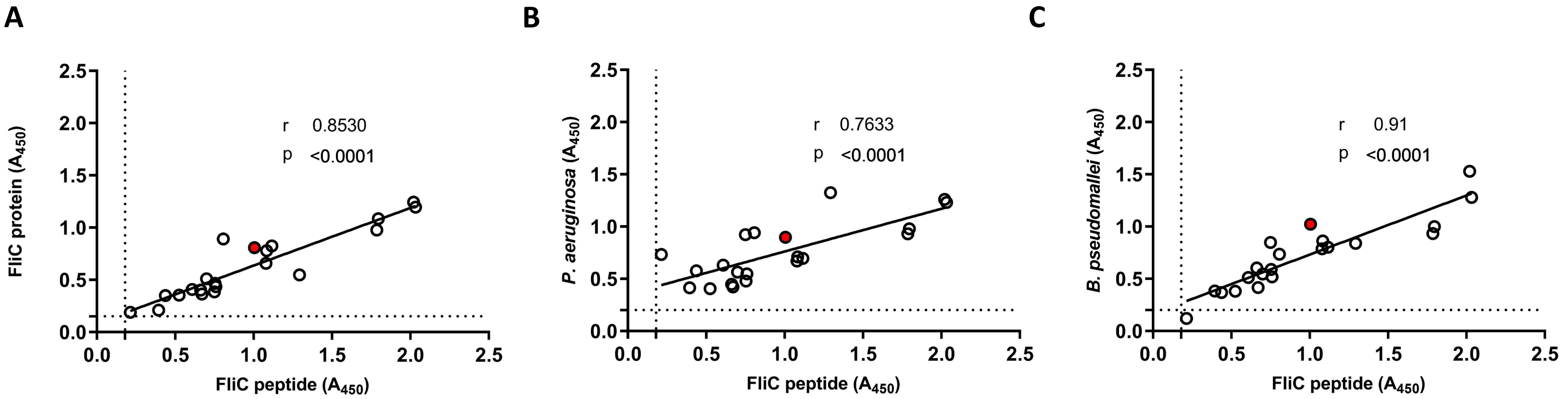

Sup Figure 1. Multi-antigen ELISA screening. Hybridoma screening by ELISA coated with the FliC peptide used in the immunization process (A-C), purified FliC protein from *P. aeruginosa* (A), whole cell *P. aeruginosa* PAO1 (B) and whole cell *B. pseudomallei* Bp82 (C). Pearson correlation analysis was used to determine the potential correlation between the two parameters.

Figure S2

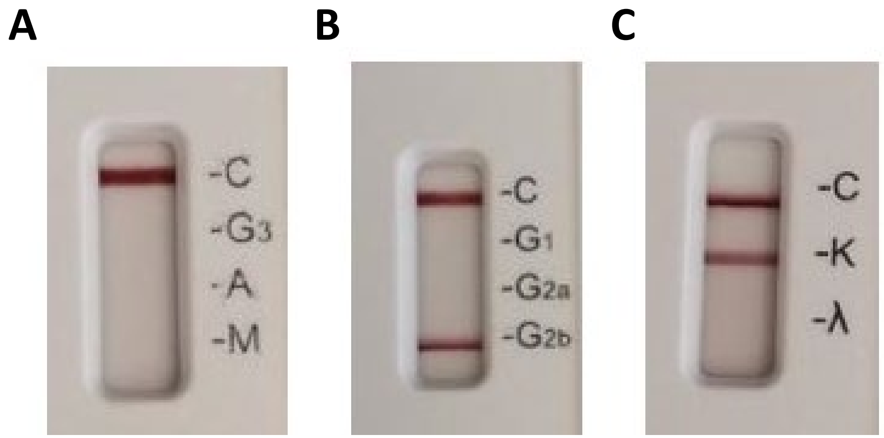

Sup Figure 2. WVDC-2109 is an IgG2bκ antibody. Results of mAb isotyping obtained with Pierce[TM] Rapid Antibody Isotyping Kit.

Figure S3

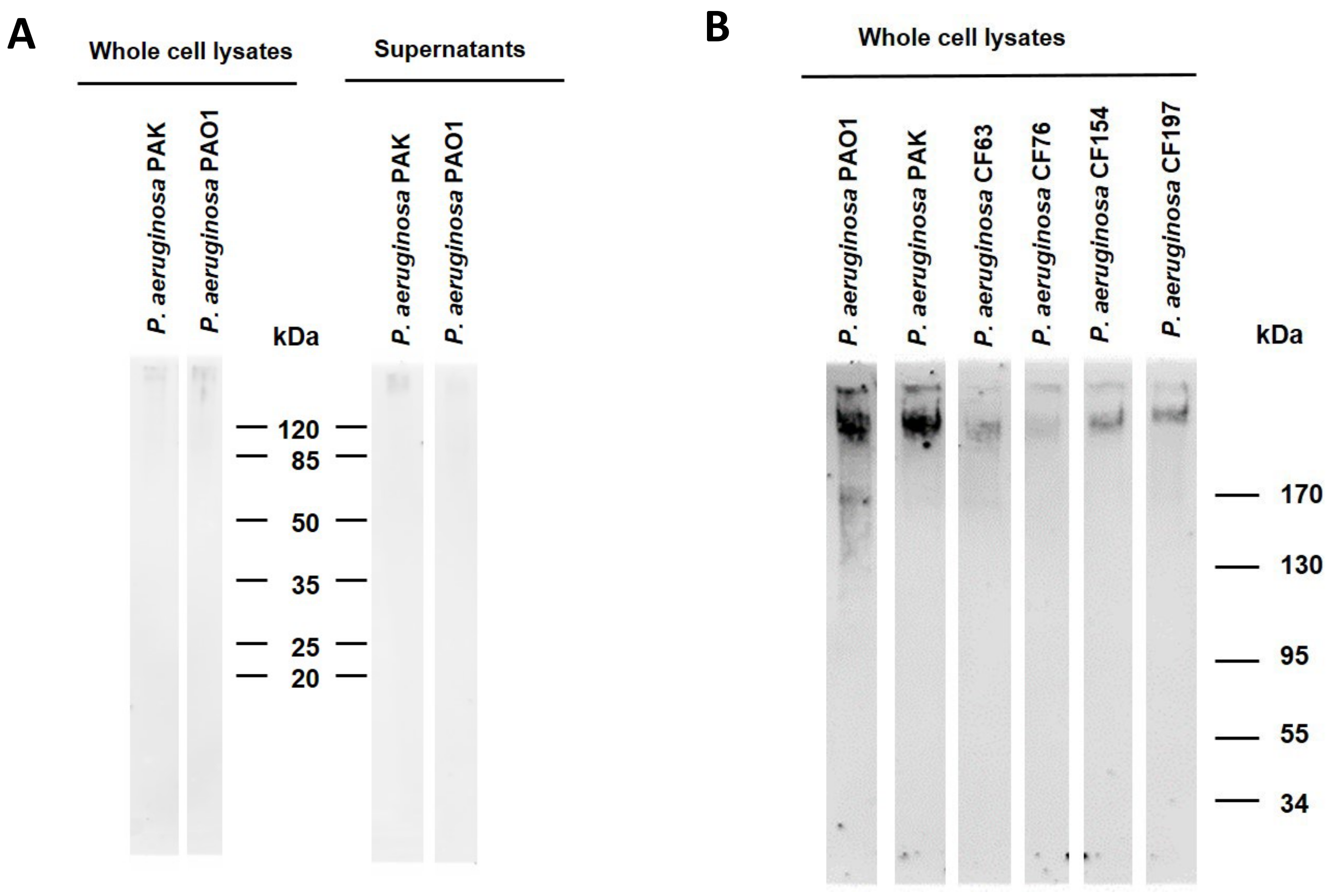

Sup Figure 3. WVDC-2109 recognizes flagellin in native conformation. (A) Representative western blot using WVDC-2109 (125 ng/ml) against whole cell lysates and cell supernatants from *P. aeruginosa* PAK (flagellin type a) and *P. aeruginosa* PAO1 (flagellin type b). (B) Representative western blot sing WVDC-2109 (625 ng/ml) against whole cell lysates from different *P. aeruginosa* strains (PAO1, PAK, CF63, CF76, CF154 and CF197). Gels were resolved using native conditions. IR 800 channel was used to capture the signals.

Figure S4

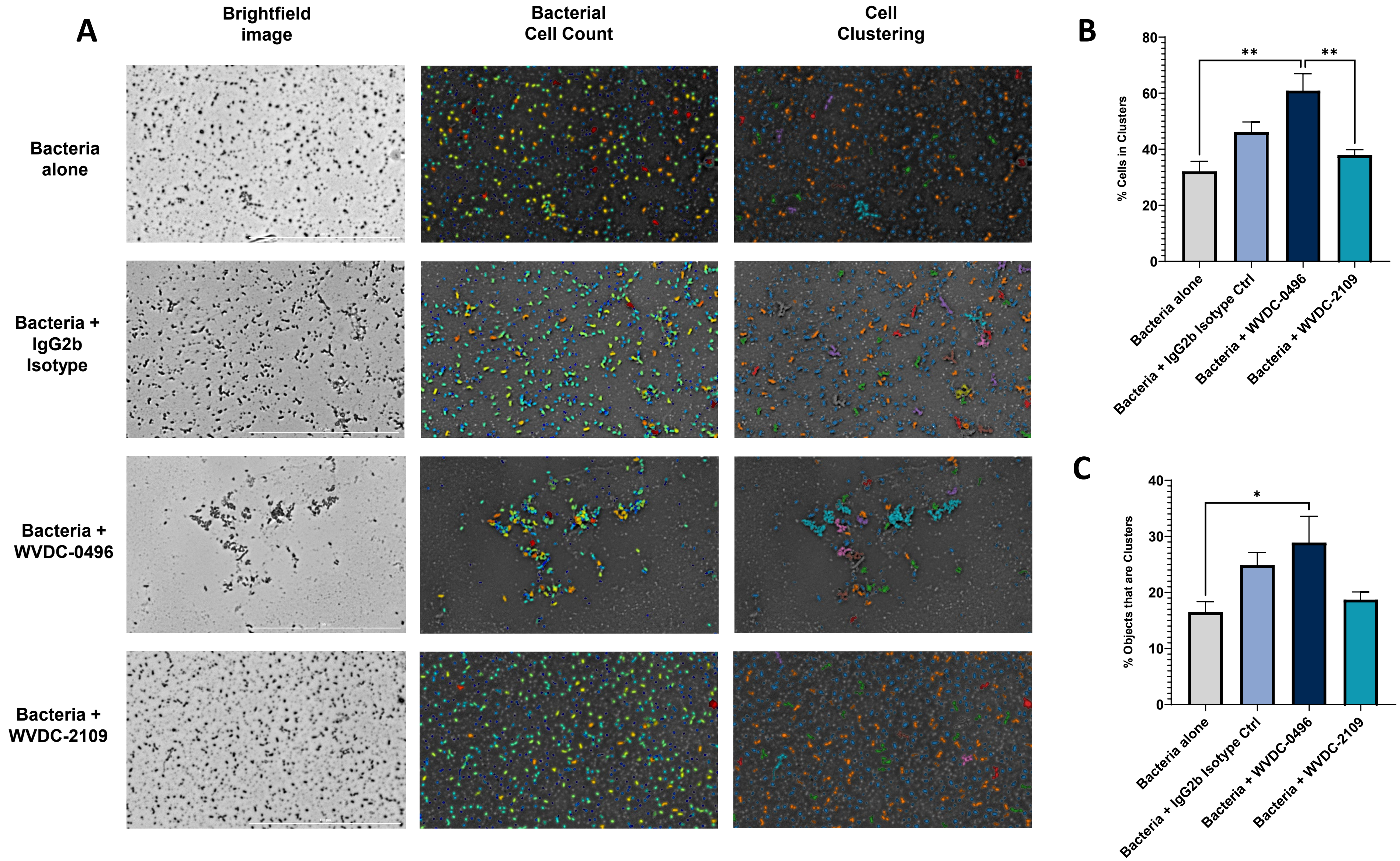

Sup Figure 4. WVDC-2109 does not induce clumping of bacterial cells. (A) Representative images obtained via Lionheart imaging under Bright field, at 60X magnification (left column) and analyzed via CellProfiler software to show the count of individual bacteria (middle column) or the amount of clustering in images (right column). Bacterial cell count images display individual cells colored by size, with separate cells colored by different colors. Cell clustering images display cells now colored by the number of cells within a cluster, with 1 cell/cluster colored as dark blue, 2 cells/cluster colored as orange, 3 cells/cluster colored as green, and so on with individual colors representing the number of cells within that cluster. (B) The percentage of cells that are in clusters per image across experimental conditions. (C) The percentage of objects that are clusters per image across experimental conditions. Statistical significance was determined by ordinary one-way ANOVA: * $p<0.05$, ** $p<0.01$. Error bars represent standard error of the mean.

Figure S5

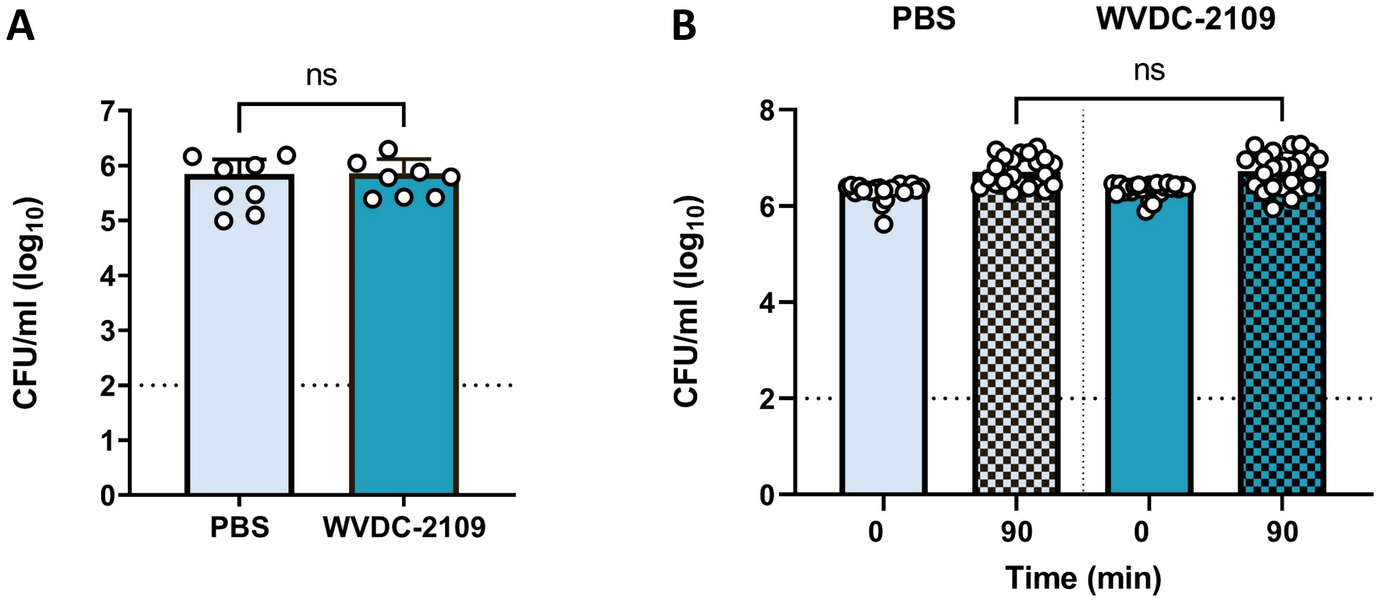

Sup Figure 5. WVDC-2109 does not increase bacterial attachment-invasion to J774A.1 cells and does not kill *P. aeruginosa* PAO1. (A) Bacterial attachment and invasion of *P. aeruginosa* PAO1 was determined by incubating J774A.1 cells and bacteria after opsonization with or without WVDC-2109. (B) Bacterial killing was determined after 60 min of incubation with WCDC-2109. Statistical significance was determined by Student’s t test. Dotted line indicates lowest limit of detection. Each dot represents one replicate. Error bars represent standard error of the mean.

Figure S6

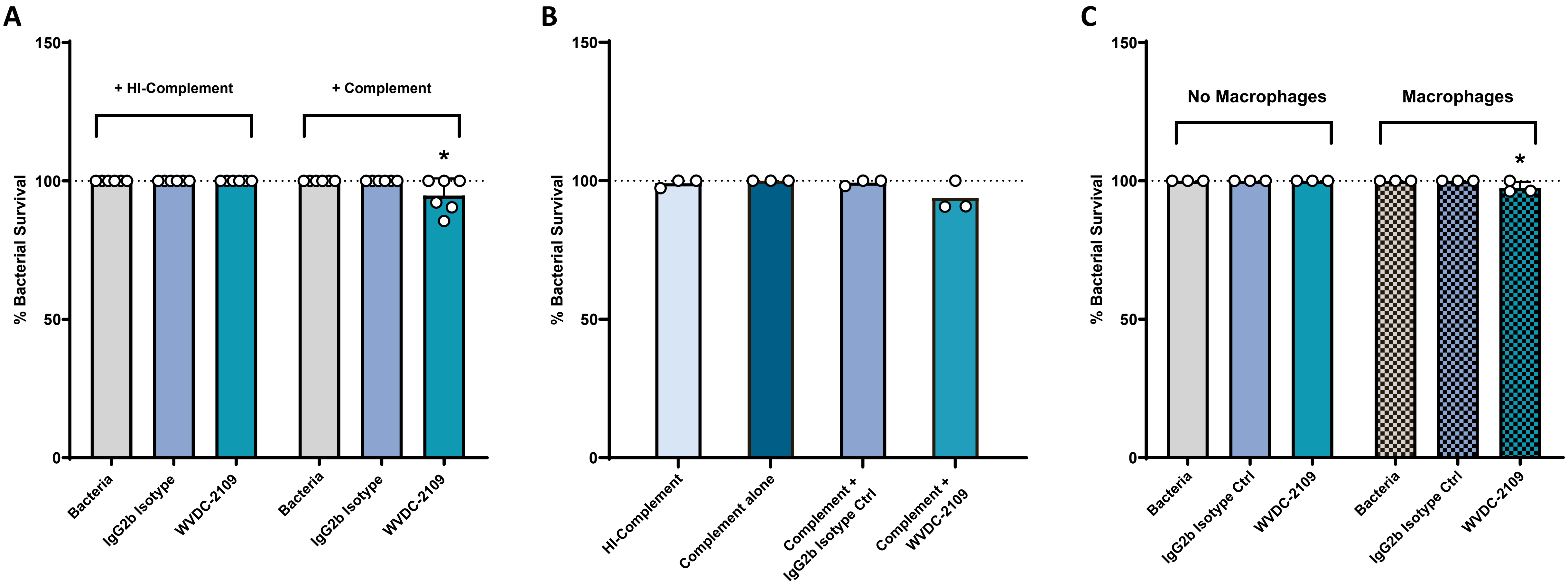

Sup Figure 6. WVDC-2109 is unable to reduce bacterial viability of PAO1 based on Complement in nutrient rich environments and does not induce bacterial mediated killing against PAK, by complement system and opsonophagocytic killing by macrophages. (A) Opsonophagocytic killing assay with addition of HI-Guinea Pig Complement or Guinea Pig Complement, in the absence of J774A.1 macrophages. Percentage of survival of *P. aeruginosa* PAO1 after 2 hours of incubation with or without WVDC-2109 or IgG2b isotype control, with added HI-Guinea Pig Complement or Guinea Pig Complement. * Denotes a comparison to all other groups. (B) Complement bactericidal assay. Percentage of survival of *P. aeruginosa* PAK after 90 min of incubation with HI-Guinea Pig Complement, Guinea Pig Complement alone, Guinea Pig Complement + IgG2b Isotype control, or Guinea Pig Complement + WVDC-2109. (C) Opsonophagocytic killing assay. Percentage of survival of *P. aeruginosa* PAK after 2 hours of incubation with macrophage J774A.1 cells previously opsonized with or without WVDC-2109 or IgG2b isotype control. * Denotes a comparison to all other groups. Statistical significance was determined by ordinary one-way ANOVA : * $p<0.05$. Dotted line represents above growth threshold. Each dot represents one replicate. Error bars represent standard error of the mean.

Figure S7

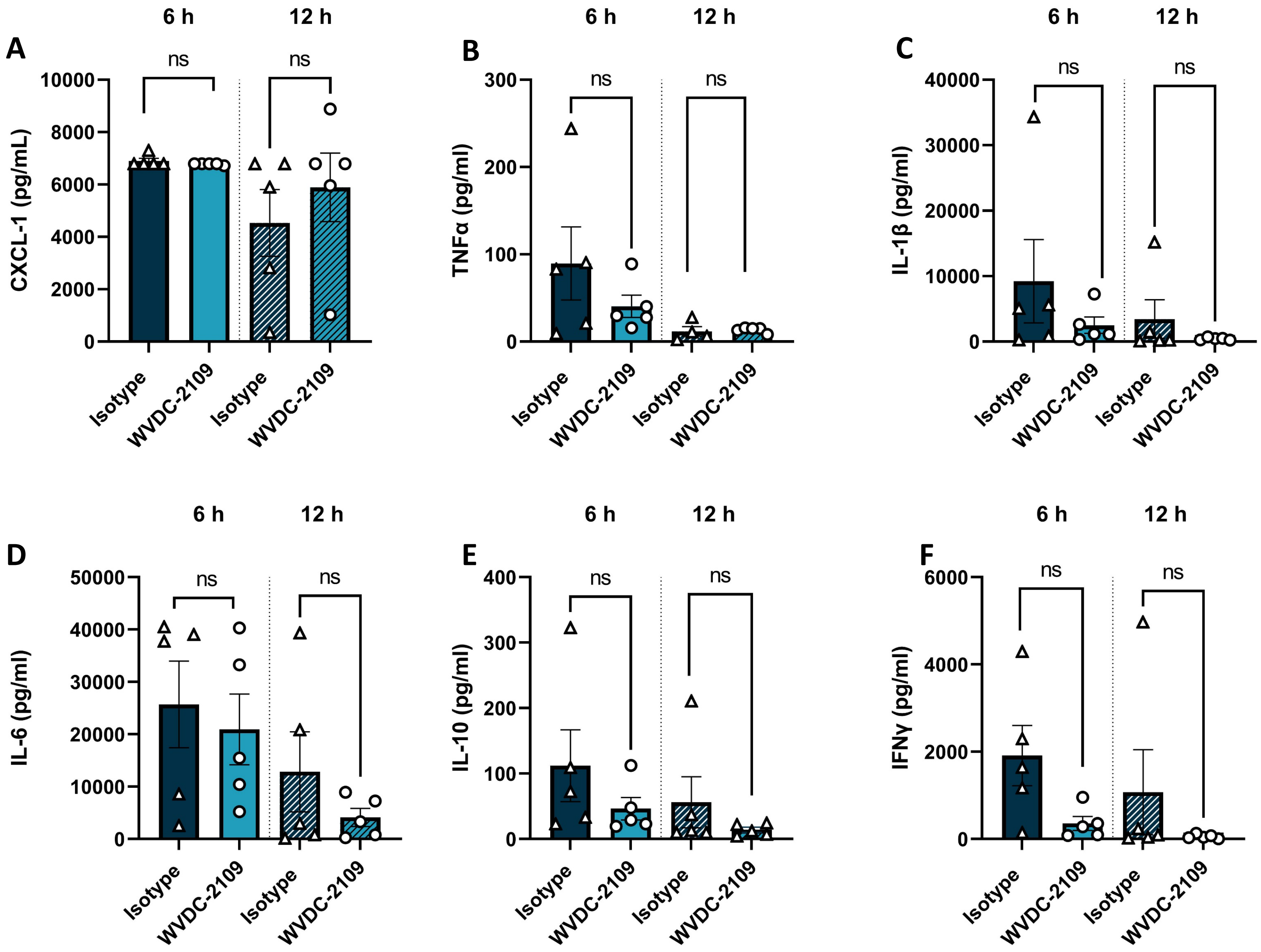

Sup Figure 7. TNFα, IL-1β, IL-6, IL-10 and IFN-γ are decreased in passively immunized WVDC-2109 mice. CXCL-1 (A), TNFα (B), IL-1β (C), IL-6 (D), IL-10 (E), IFN-γ (F) in the serum of CD-1 mice after 6 and 12 h of *P. aeruginosa* lethal infection. Mice were passively immunized with WVDC-2109 or with a non-specific IgG as a control 12 h before a *P. aeruginosa* PAO1 intraperitoneal infection.
