# Supplementary material for: Novel broadly reactive monoclonal antibody protects against Pseudomonas aeruginosa infection
Source: Infect Immun. 2024 Dec 13;93(1):e00330-24. doi: 10.1128/iai.00330-24 (PMC11784295; doi:10.1128/iai.00330-24)
Supplement: Supplemental material — Supplemental methods for Fig. S5. [file iai.00330-24-s0002.docx]

**Macrophage Bacterial Attachment assay (for Supplemental Figure 5)**

Macrophage bacterial attachment was determined by using J774A.1 (ATCC®: TIB-67) macrophages. One day before the experiment, 2.5 x 10^5^ macrophages were seeded into cell culture-treated 24-well plates while in DMEM supplemented with 10% FBS and 1% P/S. The following day, 1-2 x 10^6^ CFUs /ml of *P. aeruginosa* PAO1 from an exponential culture were opsonized with buffer alone or 100 µg/ml of WVDC-2109 for 1 h at 37ºC with slight agitation. Opsonized bacteria were then centrifuged to remove unbound antibody, resuspended in DMEM supplemented with 10% FBS and added to macrophages at multiplicity of infection of 10. Plates were centrifuged for 5 min at 250 *x* g and incubated at 33°C for 15 min and 5 % CO_2_.

To measure attachment and uptake into macrophages, plates were washed three times with PBS, and 1 ml of 0.5% Triton X-100 (Sigma-Aldrich, T8787) was added. Samples were immediately transferred into 1.5 mL tubes, serially diluted, and plated on PIA.

To measure the killing mediated by macrophages, after 15 minutes of incubation, plates were washed three times and 10 µg/ml of polymyxin B was added. Plates were incubated for 90 min at 33°C, 5% CO_2_, washed thrice, and macrophages were lysed by adding 0.5% Triton X-100. Samples were then immediately transferred into 1.5 mL tubes, serially diluted, and plated on PIA.
