# Supplementary material for: Novel broadly reactive monoclonal antibody protects against Pseudomonas aeruginosa infection
Source: Infect Immun. 2024 Dec 13;93(1):e00330-24. doi: 10.1128/iai.00330-24 (PMC11784295; doi:10.1128/iai.00330-24)
Supplement: Table S1 — Bacterial strains used in the study. [file iai.00330-24-s0004.docx]

| **Bacterial Species** | **Source** | **Notes** |
| --- | --- | --- |
| *P. aeruginosa* PAO1 | M. Vasil | Laboratory strain |
| *P. aeruginosa* PAK | S. Lory | Laboratory strain |
| *P. aeruginosa* | ATCC 15442 |  |
| *P. aeruginosa* CF8 | Burns, et al. 2001 | Collected from bronchi-alveolar lavage, serotype O9, motile |
| *P. aeruginosa* CF9 | Burns, et al. 2001 | Collected from oropharyngeal swab, serotype O5, motile |
| *P. aeruginosa* CF11 | Burns, et al. 2001 | Collected from oropharyngeal swab, serotype O1, non-motile |
| *P. aeruginosa* CF21 | Burns, et al. 2001 | Collected from oropharyngeal swab, serotype O11, non-motile |
| *P. aeruginosa* CF40 | Burns, et al. 2001 | Collected from oropharyngeal swab, serotype O6, motile |
| *P. aeruginosa* CF51 | Burns, et al. 2001 | Collected from oropharyngeal swab, serotype O1, motile |
| *P. aeruginosa* CF63 | Burns, et al. 2001 | Collected from oropharyngeal swab, serotype O1, non-motile |
| *P. aeruginosa* CF76 | Burns, et al. 2001 | Collected from oropharyngeal swab, serotype O1 |
| *P. aeruginosa* CF112 | Burns, et al. 2001 | Collected from oropharyngeal swab, serotype O6, motile |
| *P. aeruginosa* CF121 | Burns, et al. 2001 | Collected from bronchi-alveolar lavage, serotype O6, motile |
| *P. aeruginosa* CF130 | Burns, et al. 2001 | Collected from oropharyngeal swab, serotype O3, motile |
| *P. aeruginosa* CF147 | Burns, et al. 2001 | Collected from oropharyngeal swab, serotype O6, non-motile |
| *P. aeruginosa* CF154 | Burns, et al. 2001 | Collected from oropharyngeal swab, motile |
| *P. aeruginosa* CF172 | Burns, et al. 2001 | Collected from bronchi-alveolar lavage, serotype O4, motile |
| *P. aeruginosa* CF178 | Burns, et al. 2001 | Collected from oropharyngeal swab, serotype O5, motile |
| *P. aeruginosa* CF197 | Burns, et al. 2001 | Collected from bronchi-alveolar lavage, motile |
| *B. pseudomallei* Bp82 | Propst et al. 2010 | Attenuated 1026b *B. pseudomallei* strain with a partial deletion on the *purM* gene |
| *B. thailandensis* E264 | ATCC 700388 |  |
| *B. cepacia* | ATCC 25416 |  |
| *B. cenocepacia* K56-2 | P. Sokol | CF clinical isolate |
| *E. coli* | ATCC 8739 |  |
| *E. clocae* | ATCC BAA-2468 |  |
| *S. enterica* | ATCC 14028 |  |

**Sup Table 1.** Bacteria used in this study.

J. L. Burns *et al.*, “Longitudinal assessment of Pseudomonas aeruginosa in young children with cystic fibrosis,” *Journal of Infectious Diseases*, vol. 183, no. 3, 2001, doi: 10.1086/318075.

K. L. Propst, T. Mima, K. H. Choi, S. W. Dow, and H. P. Schweizer, “A Burkholderia pseudomallei ΔpurM mutant is avirulent in immunocompetent and immunodeficient animals: Candidate strain for exclusion from select-agent lists,” *Infect Immun*, vol. 78, no. 7, 2010, doi: 10.1128/IAI.01313-09.
